# Treatment Sequencing Patterns and Associated Direct Medical Costs of Metastatic Breast Cancer Care in the United States, 2011 to 2021

**DOI:** 10.1001/jamanetworkopen.2022.44204

**Published:** 2022-11-29

**Authors:** Rachel Jaber Chehayeb, Annette Hood, Xiaoliang Wang, Rebecca Miksad, Sarah Schellhorn Mougalian, Maryam B. Lustberg, Shi-Yi Wang, Rachel A. Greenup, Lajos Pusztai, Natalia Kunst

**Affiliations:** 1 Yale School of Medicine, New Haven, Connecticut; 2 Smilow Cancer Hospital, Yale New Haven Hospital, New Haven, Connecticut; 3 Flatiron Health, New York, New York; 4 Yale Cancer Center, Yale School of Medicine, New Haven, Connecticut; 5 Cancer Outcomes, Public Policy and Effectiveness Research Center, Yale University School of Medicine, New Haven, Connecticut; 6 Yale School of Public Health, New Haven, Connecticut; 7 Department of Surgery and Smilow Cancer Hospital, Yale School of Medicine, New Haven, Connecticut; 8 Department of Health Management and Health Economics, University of Oslo, Oslo, Norway; 9 Harvard Medical School & Harvard Pilgrim Health Care Institute, Boston, Massachusetts

## Abstract

**Importance:**

Advances in treatment of metastatic breast cancer (MBC) led to changes in clinical practice and treatment costs in the US over the past decade. There is limited information on current MBC treatment sequences and associated costs by MBC subtype in the US.

**Objectives:**

To identify treatment patterns by MBC subtype and associated anticancer and supportive drug costs from health care sector and Medicare perspectives.

**Design, Setting, and Participants:**

This economic evaluation analyzed data of patients with MBC obtained from the nationwide Flatiron Health database, an electronic health record–derived, deidentified database with data from community and academic practices across the US from 2011 to 2021. Participants included women aged at least 18 years diagnosed with MBC, who had at least 6 months of follow-up data, known hormone receptor (HR) and human epidermal growth factor receptor 2 (ERBB2) receptor status, and at least 1 documented line of therapy. Patients with documented receipt of clinical study drugs were excluded. Data were analyzed from June 2021 to May 2022.

**Main Outcomes and Measures:**

Outcomes of interest were frequency of different drug regimens received as a line of therapy by subtype for the first 5 lines and mean medical costs of documented anticancer treatment and supportive care drugs per patient by MBC subtype and years since metastatic diagnosis, indexed to 2021 US dollars.

**Results:**

Among 15 215 patients (10 171 patients [66.85%] with HR-positive and ERBB2-negative MBC; 2785 patients [18.30%] with HR-positive and ERBB2-positive MBC; 802 patients [5.27%] with HR-negative and ERBB2-positive MBC; 1457 patients [9.58%] with triple-negative breast cancer [TNBC]) who met eligibility criteria, 1777 (11.68%) were African American, 363 (2.39%) were Asian, and 9800 (64.41%) were White; the median (range) age was 64 (21-84) years. The mean total per-patient treatment and supportive care drug cost using publicly available Medicare prices was $334 812 for patients with HR-positive and ERBB2-positive MBC, $284 609 for patients with HR-negative and ERBB2-positive MBC, $104 774 for patients with HR-positive and ERBB2-negative MBC, and $54 355 for patients with TNBC. From 2011 to 2019 (most recent complete year 1 data are for patients diagnosed in 2019), annual costs in year 1 increased from $12 986 to $80 563 for ERBB2-negative and HR-positive MBC, $99 997 to $156 712 for ERBB2-positive and HR-positive MBC, and $31 397 to $53 775 for TNBC.

**Conclusions and Relevance:**

This economic evaluation found that drug costs related to MBC treatment increased between 2011 and 2021 and differed by tumor subtype. These findings suggest the growing financial burden of MBC treatment in the US and highlights the importance of performing more accurate cost-effectiveness analysis of novel adjuvant therapies that aim to reduce metastatic recurrence rates for early-stage breast cancer.

## Key Points

**Question:**

What are current treatment sequencing patterns for metastatic breast cancer and associated costs in the United States?

**Findings:**

In this economic evaluation of 15 215 patients between 2011 and 2021, the mean total Medicare cost of documented anticancer therapy was found to be greatest for hormone receptor–positive, human epidermal growth factor receptor 2–negative metastatic breast cancer and least for triple-negative metastatic breast cancer, with sizeable variations by subtype and year after diagnosis. Annual costs increased over time.

**Meaning:**

These findings suggest that accurate metastatic breast cancer care cost estimates are critical to assess health system financial burden of therapies and to inform cost-effectiveness analyses.

## Teaser

This economic evaluation assesses treatment patterns by metastatic breast cancer subtype and associated anticancer and supportive drug costs from health care sector and Medicare perspectives.

## Introduction

Among women in the US, breast cancer is the most diagnosed cancer and the second leading cause of cancer death, with more than 280 000 new breast cancers diagnosed each year.^1^ Although modern therapies are effective in preventing metastatic disease recurrence, approximately 15% of patients will ultimately be diagnosed with distant metastatic recurrence. An estimated 150 000 patients live with metastatic breast cancer (MBC) in the US.^2,3,4^

Over the past 5 decades, new therapies have substantially increased the median overall survival of patients with MBC from approximately 2 years to more than 5 years, depending on the molecular subtype of breast cancer, with greatest gains in hormone receptor (HR)–positive and human epidermal growth factor receptor 2 (*ERBB2*) overexpressing breast cancers.^5,6,7^ Collective strategy in the treatment of MBC is to sequence therapies until progression or unacceptable toxic effects; specific drugs depend on HR and ERBB2 status. Currently, drug selection and sequencing of therapies for MBC remains highly variable, particularly after progression on initial (ie, first line) therapy.^8^ In the past 10 years, multiple new drugs were approved to treat MBC, thus changing both treatment sequencing and costs of care.

Medical expenditures for breast cancer are highest among all cancer types with an estimated overall national cost of $26 billion in 2015 and an estimated total spending of $40.6 billion for all privately insured patients in the US in 2018.^9,10^ Estimates of MBC care cost may be less precise and are highly variable in the literature.^3,11,12,13^ Although existing literature has aimed to estimate costs associated with MBC,^3,11,12,13,14,15,16,17,18^ these cost estimates lack subgroup specificity, fail to account for recent treatment advancements, or exclude important cost components (eg, out-of-pocket costs).^3,11,12,13,14,15,17,18^ Characterization of contemporary cancer costs are critical to evaluate the financial burden faced by payers, patients, and health systems and to inform policy. Thus, we sought to determine national treatment patterns of MBC in the US, including drug regimen frequency, sequence, and duration of use, and downstream health system and Medicare costs from 2011 to 2021.

## Methods

This economic evaluation was approved by the Yale University School of Medicine institutional review board and included a waiver of informed consent because data were deidentified and this was deemed nonhuman participant research. We followed the Strengthening the Reporting of Observational Studies in Epidemiology (STROBE) reporting guideline.

### Data Source

We used the MBC cohort from the nationwide Flatiron Health electronic health record (EHR)–derived database. The Flatiron Health database is a longitudinal database, comprising deidentified patient-level structured and unstructured data, curated via technology-enabled abstraction.^19^ During the study period, the deidentified data originated from approximately 280 US cancer clinics (approximately 800 sites of care).^19^

### Study Sample

**Figure 1.**
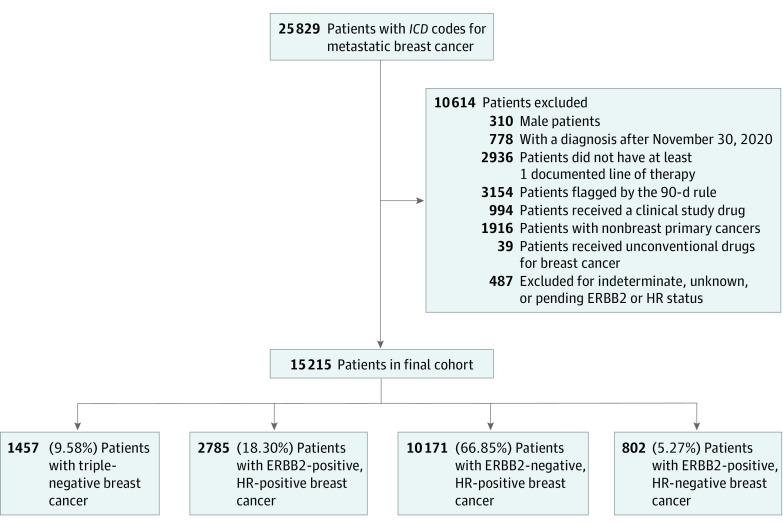
Study Flow Diagram Showing Reduction in Sample Size With the Stepwise Application of Inclusion and Exclusion Criteria ERBB2 indicates human epidermal growth factor receptor 2; HR, hormone receptor; *ICD*, *International Classification of Diseases* (the data set used *International Classification of Diseases, Ninth Revision *and *International Statistical Classification of Diseases and Related Health Problems, Tenth Revision*).

The initial data set included patients diagnosed with recurrent or de novo MBC between January 1, 2011, and May 31, 2021. We excluded patients who were male, aged younger than 18 years at metastatic diagnosis, had a documented diagnosis date within 6 months of the data cutoff date, or did not have documented therapy for MBC. We further excluded patients with more than 90 days between their MBC diagnosis and first structured documented EHR activity date because of the high risk of data incompleteness. We also excluded patients who had documentation of clinical trial drug administration, concurrent or previous *International Classification of Diseases, Ninth Revision (ICD-9)* or *International Statistical Classification of Diseases and Related Health Problems, Tenth Revision (ICD-10)* diagnosis codes for nonbreast invasive cancers, received antineoplastic drugs that are not used in the treatment of MBC, or missing, pending, or indeterminate ERBB2 and HR results (Figure 1). To assess the representativeness of our cohort in terms of clinical characteristics and demographic breakdown, we captured most recent insurance type, geographic region, age at MBC diagnosis, Eastern Cooperative Oncology Group performance status, sites of metastases, HR and ERBB2 status, body mass index, race, practice type, and drug names and administration dates. Race information provided by Flatiron Health Inc is collected from patients via intake forms or reported by clinicians through EHR workflow.

### Statistical Analysis

All analyses were performed separately for the 4 MBC subtypes. These included HR-positive and ERBB2-negative, HR-positive and ERBB2-positive, HR-negative and ERBB2-positive, and triple-negative breast cancer (TNBC).

#### Treatment Patterns

To determine treatment sequencing patterns, we tabulated the most frequently used therapies for up to 5 lines of sequential therapy. In all breast cancer subtypes, less than 15% of patients received more than 5 lines of therapy (eTable 1 and eTable 2 in Supplement 1). We excluded drug regimens that were provided to less than 0.75% of patients within a subtype. We chose 0.75% as the threshold because it captured 73% of all unique first-line therapies and limited the analysis to no more than 105 unique treatment regimens per subtype and line of therapy. We also identified the most frequent drugs received by year since MBC diagnosis.

#### Therapy Duration and Frequency and Drug Dosage

For oral drugs, we calculated treatment duration as the time between the start and end date of treatment. For non–orally administered drugs, we calculated the total number of treatment episodes based on unique drug administration dates. To avoid calculation bias in frequency and duration, we excluded patients who were still alive but had either not moved to a subsequent therapy line or had fewer than 120 days of structured activity data since their last drug episode, per Flatiron Health guidance (eMethods in Supplement 1). To calculate annual costs (cost per year since the date of MBC diagnosis), we included all patients with data and counted all anticancer therapies that were administered during the 12-month period regardless of therapy line. When treatment spanned more than 1 year, we split the duration and costs accordingly.

#### Supportive Drugs

To estimate the cost of supportive care drugs, we identified all drugs related to cancer care or to the management of adverse effects of cancer therapies. We did not consider palliative care and end-of-life costs. Identified drugs fell into 16 broad categories (eMethods in Supplement 1). We removed drugs that cost less than $100 per administration. The final list of drugs that were included in supportive care cost calculations fell into 1 of 3 categories: (1) bone marrow stimulating factors, (2) chemoprotective agents, and (3) antiemetics (eMethods in Supplement 1). We calculated the mean cumulative episode count and duration at the patient level and the cost of supportive care drugs per year and by therapy line. We then estimated a mean cost of supportive care for each MBC subtype for each therapy line and by year.

#### Cost Estimation

We used 2 sources of costs. First, we used average wholesale price (AWP) data from McKesson Corporation to calculate the costs from a health care sector perspective. We based our calculations on the price per unit and unit size per dose. Second, we used Medicare part B payment limit data for part B–covered drugs to calculate Medicare costs of each line.^20^ Given that oral drugs are not covered under Medicare part B, we used mean spending per dosage unit in 2019 provided by the Centers for Medicare & Medicaid Services Medicare Part D Drug Spending Dashboard. We indexed prices to October 2021 using the Consumer Price Index inflation calculator and applied 2021 prices to all years in the analysis.^21^ Given the lack of data on drug dosage in our data set, we used standard drug doses assuming a body surface area of 1.8 m^2^ and a weight of 80 kg. When it was not clear whether patients had received a generic or brand-name drug, we assumed that the cheaper drug was administered.

We first calculated total overall drug cost using patient-level data. Next, we calculated the mean and median price per patient per line for each regimen in each line of therapy by breast cancer subtype. For each drug regimen, we also calculated its contribution to the total price for that MBC subgroup and line of therapy. This allowed us to calculate the mean and median price of line of therapy and cost of drug treatment by year since MBC diagnosis. Finally, we stratified patients based on year of MBC diagnosis and recalculated costs to investigate how annual costs have changed with time over the past decade. All analyses were performed using R version 4.1.3 software (R Project for Statistical Computing). Data were analyzed from June 2021 to May 2022.

## Results

Our final cohort included 15 215 patients (1777 African American patients [11.68%]; 363 Asian patients [2.39%]; 9800 White patients [64.41%]), of whom 6518 (42.8%) had commercial health plans and 14 613 (96.0%) sought care in community practices (Table 1). The median (range) age was 64 (21-84) years. Overall, 10 171 (66.85%) had HR-positive and ERBB2-negative MBC, 2785 (18.30%) had HR-positive and ERBB2-positive MBC, 802 (5.27%) had HR-negative and ERBB2-positive MBC, and 1457 (9.58%) had TNBC (Figure 1).

**Table 1.** Summary of Patient Demographics and Site of Metastasis Information

Characteristic	Patients, No. (%)				
	ERBB2− and HR+ (n = 10 171)	ERBB2+ and HR+ (n = 2785)	ERBB2+ and HR− (n = 802)	TNBC (n = 1457)	All (N = 15 215)
Age at diagnosis, y^a^					
Mean (SD)	64.19 (12.15)	60.74 (12.88)	59.93 (12.68)	60.19 (12.90)	62.95 (12.51)
Median (range)	66 (23-84)	62 (21-84)	60 (24-84)	61 (24-84)	64 (21-84)
Insurance					
Commercial	4406 (43.32)	1164 (41.80)	333 (41.52)	615 (42.21)	6518 (42.84)
Other or unknown	2113 (20.77)	589 (21.15)	167 (20.82)	311 (21.35)	3180 (20.9)
Medicare	1408 (13.84)	375 (13.46)	91 (11.35)	137 (9.40)	2011 (13.22)
Patient assistance program	928 (9.12)	322 (11.56)	95 (11.85)	138 (9.47)	1483 (9.75)
Medicaid	467 (4.59)	122 (4.38)	38 (4.74)	105 (7.21)	732 (4.81)
Other government program	226 (2.22)	58 (2.08)	15 (1.87)	31 (2.13)	330 (2.17)
Self pay	68 (0.67)	24 (0.86)	10 (1.25)	14 (0.96)	116 (0.76)
Missing	555 (5.47)	131 (4.71)	53 (6.60)	106 (7.28)	845 (5.55)
Latest ECOG status					
0	2361 (23.21)	711 (25.52)	193 (24.06)	298 (20.45)	3563 (23.42)
1	2753 (27.07)	842 (30.23)	218 (27.18)	331 (22.82)	4144 (27.24)
2	1765 (17.35)	436(15.66)	125 (15.59)	264 (18.12)	2590 (17.02)
3	1043 (10.25)	264 (9.48)	74 (9.23)	183 (12.56)	1564 (10.28)
4	177 (1.74)	43 (1.54)	18 (2.24)	26 (1.78)	264 (1.74)
Missing	2072 (20.37)	489 (17.56)	174 (21.70)	355 (24.37)	3093 (20.33)
Race					
African American	1018 (10.01)	329 (11.81)	103 (12.84)	327 (22.44)	1777 (11.68)
Asian	229 (2.25)	79 (2.83)	22 (2.74)	33 (2.33)	363 (2.39)
White	6780 (66.66)	1755 (63.02)	474 (59.1)	791 (54.2)	9800 (64.41)
Other^b^	1230 (12.09)	373 (13.39)	119 (14.84)	167 (11.46)	1889 (12.41)
Missing	914 (8.99)	249 (8.94)	84 (10.47)	139 (9.47)	1386 (9.11)
Practice type					
Academic	411 (4.04)	108 (3.88)	30 (3.74)	53 (3.64)	602 (3.96)
Community	9760 (95.96)	2677 (96.12)	772 (96.26)	1404 (96.36)	14 613 (96.04)
Region					
Midwest	1585 (15.58)	390 (14.00)	94 (11.72)	195 (13.38)	2264 (14.88)
Northeast	1943 (19.10)	449 (16.12)	132 (16.46)	236 (16.20)	2760 (18.14)
South	3917 (38.51)	1186 (42.59)	356 (44.39)	668 (45.85)	6127 (40.27)
West	1806 (17.76)	523 (18.78)	143 (17.83)	217 (14.89)	2689 (17.67)
Puerto Rico	259 (2.55)	69 (2.48)	25 (3.12)	51 (3.50)	404 (2.66)
Missing	661 (6.5)	168 (6.03)	52 (6.48)	90 (6.18)	971 (6.38)
BMI					
<30	6577 (64.66)	1844 (66.21)	540 (67.33)	920 (63.14)	9881 (64.94)
>30	2848 (28.00)	742 (26.64)	200 (24.94)	364 (24.98)	4154 (29.67)
Missing	746 (7.33)	199 (7.15)	62 (7.73)	173 (11.87)	1180 (7.76)
Sites of metastasis^c^					
Bone	8002 (78.67)	1913 (68.70)	407 (50.75)	773 (53.05)	11 095 (72.92)
Liver	3651 (35.90)	1117 (40.11)	320 (39.90)	541 (37.13)	5629 (37.00)
Lung	3302 (32.46)	1056 (37.92)	351 (43.77)	800 (54.91)	5509 (36.21)
Distant lymph node	3262 (32.07)	1134 (40.72)	374 (46.63)	743 (51.00)	5513 (36.23)
Brain	1150 (11.31)	702 (25.21)	278 (34.66)	415 (28.48)	2545 (16.73)
CNS	507 (4.98)	175 (6.28)	48 (5.99)	94 (6.45)	824 (5.42)
Pleura	1505 (14.80)	323 (11.60)	73 (9.10)	231 (15.85)	2132 (14.01)
Other^d^	3341 (32.85)	821 (29.48)	316 (39.40)	493 (33.84)	4971 (32.67)

Abbreviations: −, negative; +, positive; BMI, body mass index (calculated as weight in kilograms divided by height in meters squared); CNS, central nervous system; ECOG, Eastern Cooperative Oncology Group; ERBB2, human epidermal growth factor receptor 2; HR, hormone receptor; TNBC, triple-negative breast cancer.

^a^ Patients with age greater than 85 years had their age adjusted to 85 in the Flatiron Health data set for deidentification purposes.

^b^ Other race included American Indian or Alaska Native, Hawaiian or Pacific Islander, or patients with multiple races.

^c^ Patient representation across different sites of metastases is not mutually exclusive, hence percentages within each subgroup sum up to more than 100%.

^d^ Other in metastasis includes adrenal, bone marrow, kidney, ovary, pancreas, peritoneum, skin, soft tissue, spleen, thyroid, as well as other unspecified metastases.

**Figure 2.**
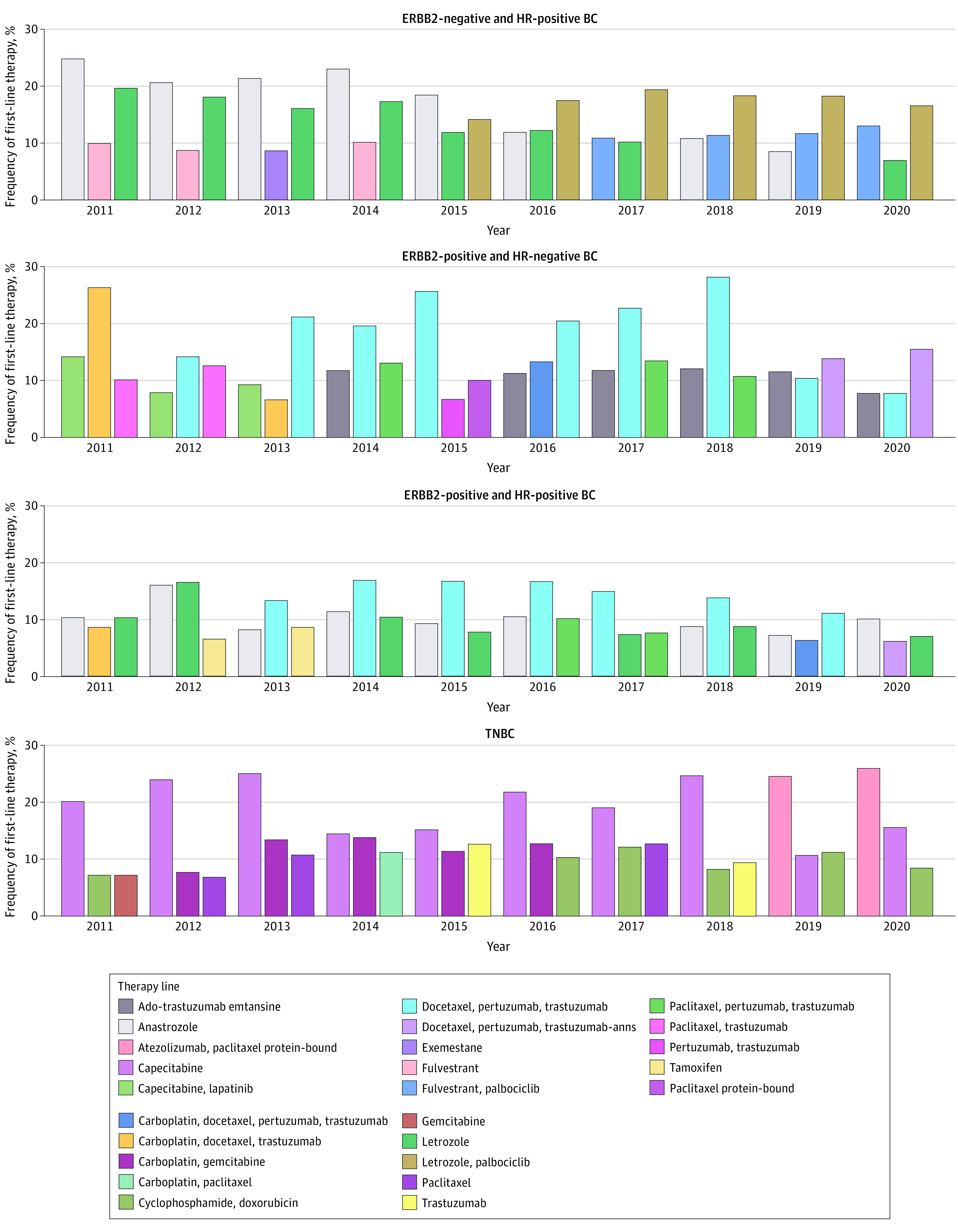
Three Most Frequent First-line Therapies by Subgroup as a Function of Calendar Year From 2011 to 2020 ERBB2 indicates human epidermal growth factor receptor 2; HR, hormone receptor; TNBC, triple-negative breast cancer.

The number of unique drug regimens documented for each line of therapy ranged between 22 and 105 across the 4 subtypes (eTable 2 in Supplement 1). The highest variation in drug regimens was seen in patients with ERBB2-positive MBC. Variation in drug regimens increased with each line of therapy. eTable 3 and eTable 4 in Supplement 1 show the most frequently used drugs for each subgroup by line of therapy and year. Across all years, the most frequent first-line therapies were anastrozole (14.52%) for HR-positive and ERBB2-negative MBC; docetaxel, pertuzumab, and trastuzumab for HR-positive and ERBB2-positive MBC (12.60%) and HR-negative and ERBB2-positive MBC (18.20%); and capecitabine (19.01%) for TNBC (eAppendix in Supplement 1). TNBC had the highest proportion of patients with documented administration of supportive care drugs across all therapy lines (eTable 5 in Supplement 1) at between 40.2% and 46.1% of patients. Figure 2 shows the change in the 3 most frequent first-line therapies by calendar year. For instance, for HR-positive and ERBB2-negative MBC, anastrozole was the most frequent first-line therapy documented in 2011, whereas the letrozole and palbociclib regimen was most frequent in 2020.

As expected, the trends in the Medicare and AWP cost estimates are parallel (eTable 6 in Supplement 1).^22^ Medicare prices are indirectly based on mean sale prices, which account for rebates and discounts but ultimately correlate with AWP and wholesale acquisition prices.^13^

The mean (SD) first-line anticancer drug costs were greatest among patients with HR-negative and ERBB2-positive MBC ($170 017 [$236 868]), followed by HR-positive and ERBB2-positive MBC ($104 072 [$159 970]) and HR-positive and ERBB2-negative MBC ($68 389 [$145 498]). Individuals with metastatic TNBC had the lowest first line anticancer drug costs ($15 633 [$43 267]). Furthermore, patients with ERBB2-positive MBC had the greatest mean anticancer therapy costs across all 5 therapy lines. The mean anticancer therapy costs decreased with each therapy line in all subgroups except for patients with TNBC, for whom the costs were constant (eTable 7 and eTable 8 in Supplement 1).

Table 2 presents mean Medicare anticancer and supportive therapy costs by year since metastatic diagnosis date for all subtypes The highest costs of treating MBC occurred in the first year after diagnosis across all subtypes. The year 1 mean (SD) anticancer therapy costs were greatest for patients with HR-positive and ERBB2-positive MBC ($131 548 [$127 805]) and patients with HR-negative and ERBB2-positive MBC ($118 062 [$96 197]), followed by patients with HR-positive and ERBB2-negative MBC ($46 712 [$57 730]). Patients with TNBC had the lowest year 1 mean (SD) anticancer therapy cost, at $26 150 ($48 822). Except for TNBC, cost by year decreased with increasing time after diagnosis. As a general trend, mean yearly supportive care drug costs and the percentage of patients receiving supportive care tended to decrease with increasing year since diagnosis (eTable 9 in Supplement 1). Furthermore, HR-negative and ERBB2-positive MBC was associated with the highest total treatment cost (ie, cost of anticancer and supportive care drugs), at $334 812, followed by HR-negative and ERBB2-positive MBC ($284 609) and HR-positive and ERBB2-negative MBC ($104 774). TNBC had the lowest total treatment cost, at $54 355. Overall, median costs were lower than means (eTable 9 and eTable 10 in Supplement 1).

**Table 2.** Annual Medicare Anticancer and Supportive Costs by Year After Diagnosis and Subgroup

Year, No.	Cost, mean (SD), 2021 $^a^			
	HR+ and ERBB2−	HR+ and ERBB2+	HR− and ERBB2+	TNBC
**Anticancer drugs**				
1	46 712 (57 730)	131 548 (127 805)	118 062 (96 187)	26 150 (48 822)
2	39 214 (50 637)	117 219 (110 026)	115 625 (78 494)	33 244 (55 952)
3	38 776 (47 238)	107 610 (108 382)	117 025 (80 768)	43 516 (63 291)
4	41 320 (53 220)	109 023 (96 450)	118 856 (89 967)	62 346 (172 839)
5	39 171 (48 296)	105 645 (88 970)	111 474 (78 176)	85 852 (172 054)
6	38 779 (46 545)	102 221 (87 178)	130 082 (73 301)	52 524 (77 622)
7	37 129 (48 385)	98 847 (80 860)	106 437 (59 636)	21 937 (16 441)
8	35 012 (44 659)	88 158 (88 649)	86 324 (66 778)	10 548 (14 870)
9	31 986 (43 401)	63 440 (62 965)	71 524 (55 226)	NA
10	31 968 (46 348)	79 695 (63 980)	25 218 (22 848)	NA
All years^b^	98 350 (114 794)	323 882 (369 573)	272 434 (311 797)	42 938 (99 523)
**Supportive drugs^c^**				
1	2967	6985	9116	8554
2	2618	2476	3278	7027
3	2567	2299	2347	7671
4	3052	2335	1725	3683
5	2681	2231	2032	3382
6	2420	1765	1882	1561
7	1820	1600	711	1820
8	1858	1989	642	NA
9	1253	1926	NA	NA
10	751	NA	NA	NA
All years	6424	10 930	12 175	11 417
**Total cost** ^c^				
All years	104 774	334 812	284 609	54 355

Abbreviations: −, negative; +, positive; ERBB2, human epidermal growth factor receptor 2; HR, hormone receptor; NA, not applicable; TNBC, triple negative breast cancer.

^a^ Means and SDs of anticancer costs for all patients in a particular subgroup and year of treatment since diagnosis, irrespective of year of diagnosis.

^b^ Note that the listed cost for all years for anticancer, supportive and total cost represent the mean cumulative cost per patient by subgroup; this cost is less than the sum of year 1 through year 10 because not all patients had 10 years of treatment data.

^c^ Given that not all patients receiving anticancer drugs in a particular subgroup and year receive supportive care drugs, the reported means reflect the ratio of the total amount spent on supportive care over the total number of patients receiving anticancer drugs in that year number and subgroup. eTable 9 in Supplement 1 shows the mean and median only for patients receiving supportive care, as well as their percentage of all patients within a subgroup by year. As such, the median supportive care cost for most years is 0, and a measure of SD would not be informative.

eTable 11 in Supplement 1 shows the drugs that were the largest contributors to overall yearly drug costs by cancer subtype and year since diagnosis, by decreasing total Medicare cost. eTable 12 in Supplement 1 shows the lines that made up the 3 largest contributors to Medicare costs by calendar year stratified by subgroup.

**Figure 3.**
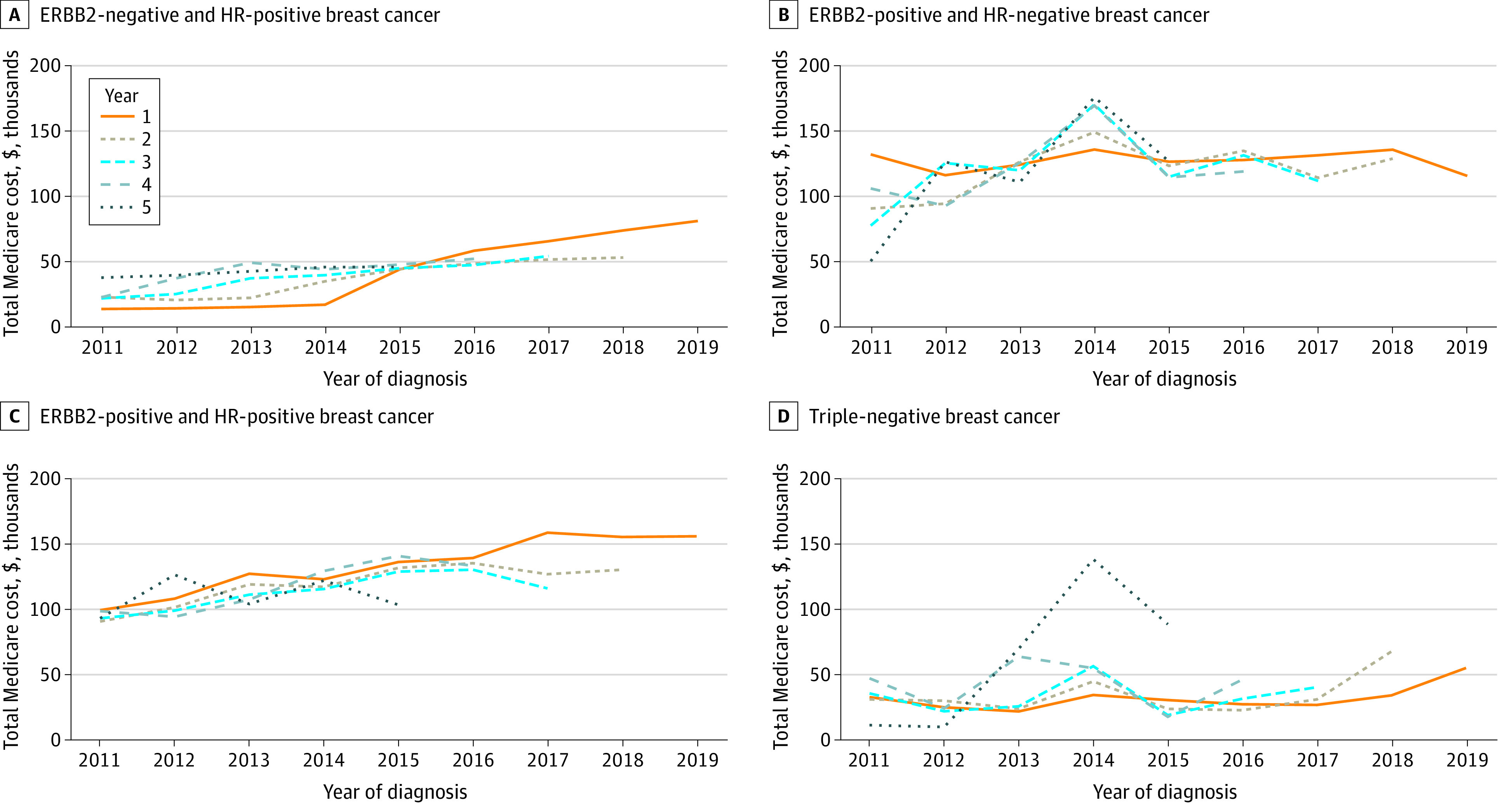
Total Mean Medicare Costs Including Both Anticancer Costs and Supportive Care Costs by Year After Diagnosis and Subgroup as a Function of Year of Diagnosis ERBB2 indicates human epidermal growth factor receptor 2; HR, hormone receptor.

We observed an increase in costs by year since diagnosis between 2011 and 2021 across most tumor subgroups (Figure 3, eTable 13 in Supplement 1). This increase was most substantial for patients with HR-positive and ERBB2-negative MBC. From 2011 to 2019 (most recent complete year 1 data are for patients diagnosed in 2019), annual treatment costs in year 1 increased from $12 986 to $80 563 for HR-positive and ERBB2-negative MBC, $99 997 to $156 712 for HR-positive and ERBB2-positive MBC, and $31 397 to $53 775 for TNBC. There was no increase in the first-year treatment cost of HR-negative and ERBB2-positive MBC, but we observed an increase in year 2 (from $90 427 to $129 690 between 2011 and 2019). Except for TNBC, supportive care costs by year after diagnosis generally decreased with increasing calendar year. Survival analysis statistics are summarized in eTable 14 and eFigure in Supplement 1.

## Discussion

To our knowledge, this economic evaluation provides the most up-to-date metastatic treatment sequences and associated anticancer and supportive therapy costs for MBC by line number and subtype in the US from 2011 until 2021. We identified large differences in cost of MBC by subtype and years since diagnosis, indicating that these factors should be taken into consideration when evaluating costs associated with treating MBC.

The increase in cost by year of diagnosis reflects the availability of novel, evolving, and more costly therapies, and improvement in survival times across the past decade; it also partially explains the great variability around mean costs, particularly for HR-positive and ERBB2-negative MBC and TNBC. Our findings also reflected differences in subtype-specific treatment costs for women with MBC.

The breakdown of our cohort by subtype is mostly in line with reported percentages from a Surveillance, Epidemiology and End Results (SEER) study of approximately 300 000 patients with MBC from 2010 to 2014 (68.1% patients with HR-positive and ERBB2-negative MBC, 9.5% patients with HR-positive and ERBB2-positive MBC, 4.1% patients with HR-negative and ERBB2-positive MBC, and 10.6% patients with TNBC).^23^ ERBB2-positive MBC was overrepresented in our more recent cohort (18.3% patients with HR-positive and ERBB2-positive MBC and 5.3% patients with HR-negative and ERBB2-positive MBC).^6^ We believe this reflects differential survival rates, with the advent of ERBB2-negative–targeted therapies, such as trastuzumab, and the lag in TNBC treatment developments, which until very recently relied on traditional chemotherapies only.^24,25,26,27,28,29^

Of note, the approval of palbociclib, ribociclib, and abemaciclib for HR-positive BC; T-DM1, neratinib, and tucatinib for ERBB2-positive BC; and olaparib for germline *BRCA* variant BC and atezolizumab (BC indication now withdrawn) took place within the study period.^25,30,31,32,33^ Our data also demonstrates the rapid adoption of palbociclib and letrozole as first-line therapy for HR-positive and ERBB2-negative MBC since the approval of palbociclib in 2015.

Decreased reliance on chemotherapies for ERBB2-positive and HR-positive subgroups can explain their decreasing supportive care cost with increasing year since diagnosis. The overall decrease in supportive care by year since diagnosis with increasing calendar year could reflect less aggressive and toxic treatments and decreased chemotherapy use with time. However, the decreasing patient count with increasing year since diagnosis limits the generalizability of these findings. Moreover, our supportive care calculations only reflect 3 main drug categories. The American Society of Clinical Oncology’s Choosing Wisely campaign could also be a potential contributory factor to both of these observed trends.^34^

Our findings on large treatment pattern heterogeneity are in line with prior research on sequential treatments. A previous study from the linked SEER and Medicare database found that 56% of patients received an overall treatment sequence that less than 11 other patients also received.^35^ In addition to treatment guidelines, tumor types, and prior treatments, insurance coverage, patient preferences, and the integration of a physician’s personal experience with current scientific literature also influence treatment choices.^35,36,37,38^ Tools are in development to rank the increasing number of regimens, such as the recently developed information theoretic network meta-analysis that was used to longitudinally rank HR-positive and ERBB2-negative treatments by efficacy.^39^

We found a large variety in drug regimens used at a line and yearly level by subgroup, with some regimens being particularly infrequent. Outlier regimens can be explained by receptor switch, concurrent illnesses, and other unique circumstances.^40^

To our knowledge, our study is the first to evaluate the medical cost of treating MBC in the US by subtype and therapy line. Previous work published in 2011 used a large private health insurance claims database with data from the 2000 to 2006 period to estimate costs of chemotherapy and supportive care in MBC but did not stratify by subgroup or line of therapy.^41^ Another retrospective study of an administrative claims database published in 2012 that covered the 2003 to 2009 period calculated mean anticancer and related treatments in per patients per month, by type of therapy received (eg, endocrine, ERBB2, cytotoxic).^42^ Our 2020 TNBC mean cost data by line of therapy are less than the estimates of a 2020 retrospective, observational study of 608 patients that estimated mean monthly costs of treatment for metastatic TNBC from 9 US community clinics.^12^ Differences in methods, populations, periods, health care settings, cohort size, price of generic vs brand name drugs, and line duration calculations can explain variations. In lieu of using patient-level data, a study published in 2012 built a MBC cost-of-illness model by subgroup in the US using results of a survey of US physicians to identify cancer treatments and SEER data for survival and incidence information.^11^ Other recent studies calculated overall costs of MBC care via matched case-control studies that compared Medicare or private insurance payments between patients with cancer and patients without cancer.^3,14^ Patient-level cost calculations are an advantage of our study, allowing for direct correlation and stratification by subgroup and line number and lending themselves well to cost-effectiveness analysis.

### Limitations

Our results should be considered in the context of several limitations. First, Medicare Part D oral drug pricing is plan-dependent, and the Part D Drug Spending Dashboard captures mean spending. Treatment costs of patients with commercial health plans (42.8% of our cohort) are also plan-dependent and not investigated in our study. Moreover, drug prices vary by region and are influenced by manufacturer discounts and rebates that we could not account for.^43^ Second, our study focused on outpatient treatment and did not account for other medical costs, such as surgery, radiation, imaging, and hospital stays. Some of these costs have been reported elsewhere.^3,18,44^ We also did not consider *BRCA* status separately.^45^ Third, we also included 2020 and 2021 drug episode data in yearly cost calculation, despite lack of complete data on first-year therapies. Hence, our yearly cost estimates are an underestimation of total costs. Importantly, our costs do not fully reflect the most recent drug approvals. Sacituzumab govitecan for TNBC, pembrolizumab plus chemotherapy for programmed cell death 1–ligand 1–positive TNBC, trastuzumab deruxtecan for low ERBB2 were approved by the US Food and Drug Administration between 2020 and 2022 and represent substantial new treatment costs.

## Conclusions

This economic evaluation found that MBC anticancer and supportive treatment costs varied by receptor subtype and time since diagnosis. Annual costs increased over time. Our study provides greater clarity around the cost breakdown of anticancer and supportive care treatment for MBC by subgroup, as well as the most common treatment sequences across the first 5 lines of therapy. Our results may help improve the evaluation of MBC treatments in the US when considering both effects and costs and emphasize the importance of considering receptor type and time since diagnosis in the evaluation.

## Notes

Supplement 1. **eMethods.** Data Source, Excluded Drugs, Modifications to Drug Tables, Supportive Drugs, and Cost Calculations **eAppendix.** Most Frequent Treatment Patterns, Regimen Information, and Survival Calculations **eTable 1.** Patient Count by Subgroup for Each Line Number **eTable 2.** Number of Drug Regimens Captured and the Corresponding Percentage of Total Drug Regimens By Subgroup and Line Number **eTable 3.** Most Frequently Used Drug Regimens at a Line Level by Percentage as a Function of Subgroup and Line Number **eTable 4.** Most Frequently Used Drugs at a Yearly Level by Patient Count Per Drug for Each Subgroup and Year **eTable 5.** Percentage of Patients Receiving Supportive Drugs by Subgroup and Line Number **eTable 6.** Annual AWP Anticancer and Supportive Drug Costs by Year After Diagnosis and Subgroup **eTable 7.** AWP and Medicare Anticancer and Supportive Drug Costs by Line and Subgroup for the First 5 Lines of Therapy **eTable 8.** Most Costly Drug Regimens by Subgroup and Associated Costs for Each of the First 5 Lines of Therapy **eTable 9.** Mean and Median Medicare Costs for Patients Receiving Supportive Care by Subgroup and Year **eTable 10.** Annual Median Medicare Anticancer Drug Costs for Patients by Year After Diagnosis and Subgroup **eTable 11.** Drugs That Made up the 3 Largest Contributors to Overall Yearly Medicare Costs Stratified by Subgroup and Year After Diagnosis **eTable 12.** Lines That Made up the 3 Largest Contributors to Medicare Costs by Calendar Year Stratified by Subgroup **eTable 13.** Mean Medicare and AWP Costs by Year After Diagnosis Stratified by Year of Diagnosis **eTable 14.** Overall Survival Statistics for the 4 Subgroups and Overall Sample **eFigure.** Overall Survival Analysis Using Kaplan-Meier Curves by Subgroup
eReferences.

Supplement 2.
Data Sharing Statement
