# Supplementary material for: Treatment Sequencing Patterns and Associated Direct Medical Costs of Metastatic Breast Cancer Care in the United States, 2011 to 2021
Source: JAMA Netw Open. 2022 Nov 29;5(11):e2244204. doi: 10.1001/jamanetworkopen.2022.44204 (PMC9709649; doi:10.1001/jamanetworkopen.2022.44204)
Supplement: Supplement 1. — eMethods. Data Source, Excluded Drugs, Modifications to Drug Tables, Supportive Drugs, and Cost Calculations eAppendix. Most Frequent Treatment Patterns, Regimen Information, and Survival Calculations eTable 1. Patient Count by Subgroup for Each Line Number eTable 2. Number of Drug Regimens Captured and the Corresponding Percentage of Total Drug Regimens By Subgroup and Line Number eTable 3. Most Frequently Used Drug Regimens at a Line Level by Percentage as a Function of Subgroup and Line Number eTable 4. Most Frequently Used Drugs at a Yearly Level by Patient Count Per Drug for Each Subgroup and Year eTable 5. Percentage of Patients Receiving Supportive Drugs by Subgroup and Line Number eTable 6. Annual AWP Anticancer and Supportive Drug Costs by Year After Diagnosis and Subgroup eTable 7. AWP and Medicare Anticancer and Supportive Drug Costs by Line and Subgroup for the First 5 Lines of Therapy eTable 8. Most Costly Drug Regimens by Subgroup and Associated Costs for Each of the First 5 Lines of Therapy eTable 9. Mean and Median Medicare Costs for Patients Receiving Supportive Care by Subgroup and Year eTable 10. Annual Median Medicare Anticancer Drug Costs for Patients by Year After Diagnosis and Subgroup eTable 11. Drugs That Made up the 3 Largest Contributors to Overall Yearly Medicare Costs Stratified by Subgroup and Year After Diagnosis eTable 12. Lines That Made up the 3 Largest Contributors to Medicare Costs by Calendar Year Stratified by Subgroup eTable 13. Mean Medicare and AWP Costs by Year After Diagnosis Stratified by Year of Diagnosis eTable 14. Overall Survival Statistics for the 4 Subgroups and Overall Sample eFigure. Overall Survival Analysis Using Kaplan-Meier Curves by Subgroup eReferences. [file jamanetwopen-e2244204-s001.pdf]

Supplemental Online Content

Jaber Chehayeb R, Hood A, Wang X, et al. Treatment sequencing patterns and associated direct medical costs of metastatic breast cancer care in the United States, 2011 to 2021. *JAMA Netw Open.* 2022;5(11):e2244204. doi:10.1001/jamanetworkopen.2022.44204

eMethods. Data Source, Excluded Drugs, Modifications to Drug Tables, Supportive Drugs, and Cost Calculations
eAppendix. Most Frequent Treatment Patterns, Regimen Information, and Survival Calculations
eTable 1. Patient Count by Subgroup for Each Line Number
eTable 2. Number of Drug Regimens Captured and the Corresponding Percentage of Total Drug Regimens By Subgroup and Line Number
eTable 3. Most Frequently Used Drug Regimens at a Line Level by Percentage as a Function of Subgroup and Line Number
eTable 4. Most Frequently Used Drugs at a Yearly Level by Patient Count Per Drug for Each Subgroup and Year
eTable 5. Percentage of Patients Receiving Supportive Drugs by Subgroup and Line Number
eTable 6. Annual AWP Anticancer and Supportive Drug Costs by Year After Diagnosis and Subgroup
eTable 7. AWP and Medicare Anticancer and Supportive Drug Costs by Line and Subgroup for the First 5 Lines of Therapy
eTable 8. Most Costly Drug Regimens by Subgroup and Associated Costs for Each of the First 5 Lines of Therapy
eTable 9. Mean and Median Medicare Costs for Patients Receiving Supportive Care by Subgroup and Year
eTable 10. Annual Median Medicare Anticancer Drug Costs for Patients by Year After Diagnosis and Subgroup
eTable 11. Drugs That Made up the 3 Largest Contributors to Overall Yearly Medicare Costs Stratified by Subgroup and Year After Diagnosis
eTable 12. Lines That Made up the 3 Largest Contributors to Medicare Costs by Calendar Year Stratified by Subgroup
eTable 13. Mean Medicare and AWP Costs by Year After Diagnosis Stratified by Year of Diagnosis
eTable 14. Overall Survival Statistics for the 4 Subgroups and Overall Sample
eFigure. Overall Survival Analysis Using Kaplan-Meier Curves by Subgroup
eReferences.

This supplemental material has been provided by the authors to give readers additional information about their work.

© 2022 Jaber Chehayeb R et al. *JAMA Network Open.*

eMethods. Data Source, Excluded Drugs, Modifications to Drug Tables, Supportive Drugs, and Cost Calculations

Data Source:

For this study, we utilized the enhanced mBC cohort database that included patient demographics (age, race, state, gender, practice type), medication orders and administrations, line of therapy, mortality, progression events, clinic visits, vital measurements, diagnoses codes, insurance status, type of practice, and sites of metastasis

Subgroup Classification:

Patients were classified as HR+ if they had any estrogen receptor positive or progesterone receptor positive tests and HER2+ if they had any HER2 test positive results (i.e., FISH positive/ amplified, NGS positive [ERRB2 amplified] or IHC positive [3+]). Patients were classified as TNBC if they were both HR- and HER2-.

Study sample: We excluded patients receiving any of the antineoplastic drugs listed that are not typically given for mBC treatment:
Temozolomide, bevacizumab, ponatinib, pazopanib, cytarabine, crizotinib, erlotinib, rituximab, gefitinib, afatinib, cetuximab, hydroxyurea, vorinostat, sorafenib, leucovorin, pomalidomide, lenalidomide, tretinoin, osimertinib, chlorambucil, ibrutinib, trifluridine/tipiracil, regorafenib, venetoclax, ruxolitinib, imatinib, dasatinib, lenvatinib, ixazomib, levoleucovorin, sunitinib, larotrectinib, mesna, bortezomib, cabozantinib, cobimetinib, melphalan, tazemetostat.

Censoring: Per Flatiron Health guidance, to consider a line of therapy completed, one of three conditions had to be met: (1) initiation of a new line of therapy, (2) death while receiving therapy, or (3) presence of a gap of >120 days between the last administration date and last visit/ structured activity date. Any patients that did not meet these criteria were censored when calculating mean line of therapy costs. No equivalent censoring was done at the yearly level.

Treatment Patterns:

The following modifications were made during patient-level drug episode analysis:

- Medications for which calculated duration was zero were excluded i.e., start date and end date were identical (143 out of 31,807 drug entries).
- Some drug listings had a large discrepancy between the line of therapy duration and drug (1,009 out of 33,440 overall drugs and out of 12,831 oral drug episodes). We excluded cases with the oral drug duration below a quarter of the duration of therapy line, given the high chance of potential errors and underestimation of the true medical duration in these cases.
- For ordered non-cancelled oral drugs with no abstraction dates, duration on the drug was considered equal to the duration of other therapies in the same regimen or to the duration of the line of therapy if there were no other concurrent drugs given.
- Loading dose costs were added for drugs in which loading doses are administered in clinical practice: (trastuzumab-anns, trastuzumab-qyyp, trastuzumab-dkst, trastuzumab-dttb, trastuzumab-pkrb, pertuzumab).
- For carboplatin and paclitaxel, we calculated the percentage of total patients receiving high (once every three weeks) and low (once weekly) doses and factored those percentages in the price calculations.
- For all the filgrastim generics, we calculated the percentage of patients receiving each of high dose (480 mg) and low dose (300mg) and incorporated those percentages into cost calculations. For tbo-filgrastim, 21% of patients received low dose. For aafi-filgrastim, 25% of patients received the low dose. For sndz-filgrastim, 39% of patients received the low dose. For filgrastrim, 43% of patients received the low dose.
- We assumed all patients who received oral aprepitant received it in the tri-pack formulation

Supportive Drugs:

© 2022 Jaber Chehayeb R et al. *JAMA Network Open.*

- To estimate the cost of supportive care drugs, we identified all non-cancelled medication orders/medication administrations and removed anti-cancer drugs and drugs generally unrelated to cancer care or to the management of adverse effects of cancer therapies. The remaining drugs (non-endocrine or anti-neoplastic) fell into 16 broad categories: steroid, antiemetic, hematological agent, proton pump inhibitor, antidepressant, antidote and reversal agent, solution-fluid, bone therapy agent, pain agent, anesthetic, anticholinergic, gout, and hyperuricemia agent, cytoprotective, anti-infective, antihyperglycemic, sedative agent.
- Anti-coagulants and anti-platelets were not included in supportive drug calculations.
- The supportive drugs that were included in cost calculations are dexrazoxane, pegfilgrastim (Neulasta) and generics, palonosetron, aprepitant, fosaprepitant, filgrastim (Neopogen) and generics, granisetron (including Sustol and Sancuso), rolapitant, netupitant/palonosetron HCl, and fosnetupitant/palonosetron HCl.
  The pegfilgrastim generics we considered are Fulphila, Nyvepria, Udenyca, and Ziextenzo.
- The filgrastim generics we considered are Granix, Nivestym and Zarxio.

Cost calculations:

- We used 2021 AWP pricing as a stand-in for Medicare part D pricing for tucatinib since the drug was not approved yet in 2019.
- We used standard oral drug frequencies for cost calculation purposes.
  We considered neratinib, letrozole, talazoparib, anastrozole, everolimus, tamoxifen, lapatinib, alpeslisib, and exemstane to be taken once daily, continuously, and olaparib, abemacicilib, and tucatinib to be taken twice daily, continuously. We considered capecitabine to be taken twice daily, two weeks on and one week off, and palbocicilb and ribocicilib to be taken once daily, three weeks on and one week off.

Survival Analysis

To validate our data, we performed a survival analysis to examine whether the overall survival of the patients included in our dataset followed the survival trends in the published literature. We performed this analysis following Flatiron Health methodology guidance and estimated survival for the overall cohort and for each of the 4 mBC subgroups. Patients who were still alive were censored at their last structured activity date (i.e., visit or medication order/ administration). The analyses were performed using R software and packages ‘Survival’ and ‘survminer.’

© 2022 Jaber Chehayeb R et al. *JAMA Network Open.*

eAppendix. Most Frequent Treatment Patterns, Regimen Information, and Survival Calculations

We differentiated between two dosing strategies for paclitaxel and carboplatin that are associated with different costs. We calculated that 24% of patients received high dose paclitaxel once every 3 weeks while 76 % received low dose paclitaxel once per week. For carboplatin, 60% of patients received high doses every three weeks whereas 40% of patients received a low dose once a week.

Among patients with HR+HER2- mBC, the most frequent first line therapy was anastrozole (14.52%) and second line therapy was fulvestrant (10.62%). Capecitabine was the most frequently used drug in the third therapy line (9.62%), fourth therapy line (11.06%,), and fifth therapy line (9.20%). Among patients with HR+HER2+ mBC, the most frequent first line therapy was the drug regimen consisting of docetaxel, pertuzumab, trastuzumab (12.60%). Ado-trastuzumab emtansine was the most frequent drug for therapy lines 2 through 5, making up 8.80% of second line regimens, 9.61 % of third line regimens, 9.06% of fourth line regimens and 8.51% of fifth line regimens. Among patients with HR-HER2+ mBC, the most frequent first line regimen was docetaxel, pertuzumab, and trastuzumab (18.20%). Ado-trastuzumab emtansine was the most frequent second, (32.92 %), third (16.36%) and fourth (12.39%) line of therapy (eTable3). Further, the drug regimen consisting of doxorubicin, pegylated and liposomal was the most common fifth therapy line (8.06%). Among patients with TNBC, capecitabine was the most frequent first (19.01%) and second line therapy (13.94%), while eribulin was the most frequent third (18.53%), fourth (14.65%) and fifth line of therapy (10.98%).

eTable 4 shows the five most frequently received individual drugs by year since date of metastatic diagnosis, as defined by highest patient count per year and subgroup. For year 1, the most frequently used drugs are palbociclib (n=3362 patients, HR+HER2-), trastuzumab (n=1595 patients, HR+HER2+ and n=566 patients, HR-HER2+ and capecitabine (n=448 patients, TNBC).

Survival calculations: Patients with HR+HER2+ mBC had the highest median survival (37.6 months), followed by patients with HR+HER2- mBC (32.3 months), patients with HR-HER2+ mBC (20.9 months), and patients with TNBC (10.44 months; eTable 12, eFigure 1). These overall survival times agree with estimates from the literature sample, albeit trending slightly lower[1-7].

© 2022 Jaber Chehayeb R et al. *JAMA Network Open.*

eTable 1. Patient Count by Subgroup for Each Line Number

	Line of Therapy Number																		
**Subgroup**	**1**	**2**	**3**	**4**	**5**	**6**	**7**	**8**	**9**	**10**	**11**	**12**	**13**	**14**	**15**	**16**	**17**	**18**	**% with >5**
**TNBC**	1457	775	394	198	82	37	17	8	4	2	0	0	0	0	0	0	0	0	2.5
**HER2+/HR-**	802	407	220	113	62	44	24	10	4	3	0	0	0	0	0	0	0	0	5.5
**HER2+/HR+**	2785	2034	1332	861	552	349	226	133	76	37	19	7	3	1	1	0	0	0	12.5
**HER2-/HR+**	10171	6546	4179	2640	1676	1046	639	382	204	101	58	27	14	6	2	2	1	1	10.3

*Given that receiving at least one line of therapy was among our inclusion criteria, the number receiving LOT#1 is equal to the total number of patients in that subset.

© 2022 Jaber Chehayeb R et al. *JAMA Network Open.*

eTable 2. Number of Drug Regimens Captured and the Corresponding Percentage of Total Drug Regimens By Subgroup and Line Number

Subgroup	Line Number	Total Percent Covered (%)	Number of Regimens
**HR+HER2-**	1	80.8	47
**HR+HER2-**	2	72.4	49
**HR+HER2-**	3	70.6	50
**HR+HER2-**	4	67.6	28
**HR+HER2-**	5	69.2	37
**HR+HER2+**	1	72.9	71
**HR+HER2+**	2	58.8	93
**HR+HER2+**	3	58.9	81
**HR+HER2+**	4	55.1	72
**HR+HER2+**	5	50.7	47
**HR-HER2+**	1	80.5	69
**HR-HER2+**	2	74.4	44
**HR-HER2+**	3	83.6	74
**HR-HER2+**	4	100	105
**HR-HER2+**	5	100	77
**TNBC**	1	85.2	31
**TNBC**	2	80.5	29
**TNBC**	3	84.5	30
**TNBC**	4	80.8	22
**TNBC**	5	100	46

*We filtered out all drug combinations that less than 0.75% of patients in a subgroup received for a particular line.

© 2022 Jaber Chehayeb R et al. *JAMA Network Open.*

eTable 3. Most Frequently Used Drug Regimens at a Line Level by Percentage as a Function of Subgroup and Line Number

Subgroup	LineName	Percent (%)	Line Number
**HR+HER2-**	Anastrozole	14.52	1
**HR+HER2-**	Letrozole	12.05	1
**HR+HER2-**	Letrozole, Palbociclib	12.05	1
**HR+HER2-**	Fulvestrant	6.79	1
**HR+HER2-**	Fulvestrant, Palbociclib	6.53	1
**HR+HER2-**	Tamoxifen	5.78	1
**HR+HER2-**	Exemestane	3.88	1
**HR+HER2-**	Capecitabine	3.25	1
**HR+HER2-**	Cyclophosphamide,Doxorubicin	3.06	1
**HR+HER2-**	Paclitaxel Protein-Bound	2.48	1
**HR+HER2-**	Paclitaxel	2.24	1
**HR+HER2-**	Anastrozole,Palbociclib	1.75	1
**HR+HER2-**	Letrozole,Ribociclib	1.37	1
**HR+HER2-**	Cyclophosphamide,Docetaxel	1.3	1
**HR+HER2-**	Carboplatin,Gemcitabine	1.04	1
**HR+HER2-**	Fulvestrant	10.62	2
**HR+HER2-**	Fulvestrant,Palbociclib	9.49	2
**HR+HER2-**	Letrozole,Palbociclib	7.32	2
**HR+HER2-**	Capecitabine	6.31	2
**HR+HER2-**	Everolimus,Exemestane	4.63	2
**HR+HER2-**	Paclitaxel	4.51	2
**HR+HER2-**	Letrozole	3.54	2
**HR+HER2-**	Exemestane	3.44	2
**HR+HER2-**	Anastrozole	3.07	2
**HR+HER2-**	Tamoxifen	2.86	2
**HR+HER2-**	Paclitaxel Protein-Bound	2.52	2
**HR+HER2-**	Anastrozole,Fulvestrant	1.88	2
**HR+HER2-**	Abemaciclib,Fulvestrant	1.57	2
**HR+HER2-**	Carboplatin,Gemcitabine	1.54	2
**HR+HER2-**	Eribulin	1.53	2
**HR+HER2-**	Gemcitabine	1.42	2
**HR+HER2-**	Fulvestrant,Letrozole	1.41	2
**HR+HER2-**	Anastrozole,Palbociclib	1.39	2
**HR+HER2-**	Capecitabine	9.62	3

© 2022 Jaber Chehayeb R et al. *JAMA Network Open.*

Subgroup	LineName	Percent (%)	Line Number
**HR+HER2-**	Fulvestrant,Palbociclib	7.23	3
**HR+HER2-**	Everolimus,Exemestane	6.39	3
**HR+HER2-**	Fulvestrant	6.1	3
**HR+HER2-**	Letrozole,Palbociclib	4.16	3
**HR+HER2-**	Eribulin	3.95	3
**HR+HER2-**	Paclitaxel Protein-Bound	3.66	3
**HR+HER2-**	Paclitaxel	2.92	3
**HR+HER2-**	Tamoxifen	2.92	3
**HR+HER2-**	Letrozole	2.9	3
**HR+HER2-**	Gemcitabine	2.8	3
**HR+HER2-**	Exemestane	2.68	3
**HR+HER2-**	Anastrozole	2.58	3
**HR+HER2-**	Doxorubicin Pegylated Liposomal	1.7	3
**HR+HER2-**	Alpelisib,Fulvestrant	1.68	3
**HR+HER2-**	Abemaciclib,Fulvestrant	1.63	3
**HR+HER2-**	Carboplatin,Gemcitabine	1.58	3
**HR+HER2-**	Vinorelbine	1.1	3
**HR+HER2-**	Capecitabine	11.06	4
**HR+HER2-**	Eribulin	6.63	4
**HR+HER2-**	Everolimus,Exemestane	5.76	4
**HR+HER2-**	Fulvestrant,Palbociclib	5.64	4
**HR+HER2-**	Paclitaxel Protein-Bound	5.53	4
**HR+HER2-**	Fulvestrant	4.39	4
**HR+HER2-**	Gemcitabine	3.86	4
**HR+HER2-**	Letrozole,Palbociclib	3.56	4
**HR+HER2-**	Paclitaxel	3.52	4
**HR+HER2-**	Doxorubicin Pegylated Liposomal	3.03	4
**HR+HER2-**	Vinorelbine	2.01	4
**HR+HER2-**	Tamoxifen	1.97	4
**HR+HER2-**	Exemestane	1.74	4
**HR+HER2-**	Carboplatin,Gemcitabine	1.59	4
**HR+HER2-**	Alpelisib,Fulvestrant	1.29	4
**HR+HER2-**	Anastrozole	1.29	4
**HR+HER2-**	Capecitabine,Fulvestrant	1.25	4
**HR+HER2-**	Abemaciclib,Fulvestrant	1.21	4

© 2022 Jaber Chehayeb R et al. *JAMA Network Open.*

Subgroup	LineName	Percent (%)	Line Number
**HR+HER2-**	Letrozole	1.17	4
**HR+HER2-**	Docetaxel	1.06	4
**HR+HER2-**	Capecitabine	9.19	5
**HR+HER2-**	Eribulin	7.52	5
**HR+HER2-**	Gemcitabine	7.34	5
**HR+HER2-**	Paclitaxel Protein-Bound	4.65	5
**HR+HER2-**	Everolimus,Exemestane	4.36	5
**HR+HER2-**	Paclitaxel	4.3	5
**HR+HER2-**	Fulvestrant,Palbociclib	3.82	5
**HR+HER2-**	Doxorubicin Pegylated Liposomal	3.7	5
**HR+HER2-**	Vinorelbine	3.04	5
**HR+HER2-**	Letrozole,Palbociclib	2.74	5
**HR+HER2-**	Fulvestrant	2.68	5
**HR+HER2-**	Carboplatin,Gemcitabine	2.09	5
**HR+HER2-**	Alpelisib,Fulvestrant	1.67	5
**HR+HER2-**	Docetaxel	1.25	5
**HR+HER2-**	Tamoxifen	1.19	5
**HR+HER2-**	Abemaciclib,Fulvestrant	1.13	5
**HR+HER2-**	Cyclophosphamide,Doxorubicin	1.13	5
**HR+HER2-**	Exemestane	1.01	5
**HR+HER2-**	Ixabepilone	1.01	5
**HR-HER2+**	Docetaxel,Pertuzumab,Trastuzumab	18.2	1
**HR-HER2+**	Ado-Trastuzumab Emtansine	8.1	1
**HR-HER2+**	Paclitaxel,Pertuzumab,Trastuzumab	7.73	1
**HR-HER2+**	Trastuzumab	6.23	1
**HR-HER2+**	Carboplatin,Docetaxel,Pertuzumab,Trastuzumab	5.74	1
**HR-HER2+**	Paclitaxel,Trastuzumab	4.61	1
**HR-HER2+**	Capecitabine,Lapatinib	4.11	1
**HR-HER2+**	Carboplatin,Docetaxel,Trastuzumab	3.62	1
**HR-HER2+**	Capecitabine	3.49	1
**HR-HER2+**	Pertuzumab,Trastuzumab	3.12	1
**HR-HER2+**	Docetaxel,Pertuzumab,Trastuzumab-Anns	2.87	1
**HR-HER2+**	Cyclophosphamide,Doxorubicin	1.75	1
**HR-HER2+**	Trastuzumab,Vinorelbine	1.75	1
**HR-HER2+**	Docetaxel,Paclitaxel,Pertuzumab,Trastuzumab	1.37	1

© 2022 Jaber Chehayeb R et al. *JAMA Network Open.*

Subgroup	LineName	Percent (%)	Line Number
**HR-HER2+**	Lapatinib	1.12	1
**HR-HER2+**	Paclitaxel	1.12	1
**HR-HER2+**	Paclitaxel Protein-Bound,Trastuzumab	1.12	1
**HR-HER2+**	Carboplatin,Gemcitabine	1	1
**HR-HER2+**	Ado-Trastuzumab Emtansine	32.92	2
**HR-HER2+**	Capecitabine,Lapatinib	4.91	2
**HR-HER2+**	Docetaxel,Pertuzumab,Trastuzumab	3.44	2
**HR-HER2+**	Trastuzumab,Vinorelbine	2.95	2
**HR-HER2+**	Capecitabine	2.7	2
**HR-HER2+**	Eribulin	2.7	2
**HR-HER2+**	Lapatinib,Trastuzumab	2.7	2
**HR-HER2+**	Pertuzumab,Trastuzumab	2.7	2
**HR-HER2+**	Paclitaxel,Pertuzumab,Trastuzumab	2.46	2
**HR-HER2+**	Capecitabine,Trastuzumab	2.21	2
**HR-HER2+**	Paclitaxel,Trastuzumab	1.97	2
**HR-HER2+**	Pertuzumab,Trastuzumab,Vinorelbine	1.72	2
**HR-HER2+**	Trastuzumab	1.72	2
**HR-HER2+**	Capecitabine,Lapatinib,Trastuzumab	1.47	2
**HR-HER2+**	Cyclophosphamide,Doxorubicin	1.47	2
**HR-HER2+**	Capecitabine,Trastuzumab-Anns,Tucatinib	1.23	2
**HR-HER2+**	Gemcitabine	1.23	2
**HR-HER2+**	Ado-Trastuzumab Emtansine	16.36	3
**HR-HER2+**	Capecitabine,Lapatinib	6.36	3
**HR-HER2+**	Fam-Trastuzumab Deruxtecan-Nxki	5.45	3
**HR-HER2+**	Capecitabine	4.09	3
**HR-HER2+**	Eribulin	3.64	3
**HR-HER2+**	Gemcitabine,Trastuzumab	3.64	3
**HR-HER2+**	Docetaxel,Pertuzumab,Trastuzumab	2.73	3
**HR-HER2+**	Lapatinib,Trastuzumab	2.73	3
**HR-HER2+**	Capecitabine,Trastuzumab	2.27	3
**HR-HER2+**	Eribulin,Trastuzumab	2.27	3
**HR-HER2+**	Paclitaxel,Pertuzumab,Trastuzumab	2.27	3
**HR-HER2+**	Trastuzumab	2.27	3
**HR-HER2+**	Capecitabine,Neratinib	1.82	3
**HR-HER2+**	Capecitabine,Trastuzumab-Anns,Tucatinib	1.82	3

© 2022 Jaber Chehayeb R et al. *JAMA Network Open.*

Subgroup	LineName	Percent (%)	Line Number
**HR-HER2+**	Lapatinib	1.82	3
**HR-HER2+**	Paclitaxel Protein-Bound	1.82	3
**HR-HER2+**	Paclitaxel Protein-Bound,Trastuzumab	1.82	3
**HR-HER2+**	Trastuzumab,Vinorelbine	1.82	3
**HR-HER2+**	Capecitabine,Pertuzumab,Trastuzumab	1.36	3
**HR-HER2+**	Cyclophosphamide,Doxorubicin	1.36	3
**HR-HER2+**	Doxorubicin Pegylated Liposomal	1.36	3
**HR-HER2+**	Pertuzumab,Trastuzumab	1.36	3
**HR-HER2+**	Pertuzumab,Trastuzumab,Vinorelbine	1.36	3
**HR-HER2+**	Ado-Trastuzumab Emtansine	12.39	4
**HR-HER2+**	Capecitabine,Lapatinib	7.08	4
**HR-HER2+**	Eribulin	5.31	4
**HR-HER2+**	Fam-Trastuzumab Deruxtecan-Nxki	5.31	4
**HR-HER2+**	Trastuzumab,Vinorelbine	4.42	4
**HR-HER2+**	Capecitabine,Trastuzumab-Anns,Tucatinib	3.54	4
**HR-HER2+**	Doxorubicin Pegylated Liposomal	3.54	4
**HR-HER2+**	Gemcitabine,Trastuzumab	3.54	4
**HR-HER2+**	Gemcitabine	2.65	4
**HR-HER2+**	Pertuzumab,Trastuzumab,Vinorelbine	2.65	4
**HR-HER2+**	Vinorelbine	2.65	4
**HR-HER2+**	Capecitabine	1.77	4
**HR-HER2+**	Capecitabine,Trastuzumab	1.77	4
**HR-HER2+**	Capecitabine,Trastuzumab,Tucatinib	1.77	4
**HR-HER2+**	Docetaxel,Pertuzumab,Trastuzumab	1.77	4
**HR-HER2+**	Eribulin,Trastuzumab	1.77	4
**HR-HER2+**	Gemcitabine,Pertuzumab,Trastuzumab	1.77	4
**HR-HER2+**	Ixabepilone	1.77	4
**HR-HER2+**	Lapatinib	1.77	4
**HR-HER2+**	Lapatinib,Trastuzumab	1.77	4
**HR-HER2+**	Sacituzumab Govitecan-Hziy	1.77	4
**HR-HER2+**	Trastuzumab	1.77	4
**HR-HER2+**	Doxorubicin Pegylated Liposomal	8.06	5
**HR-HER2+**	Ado-Trastuzumab Emtansine	6.45	5
**HR-HER2+**	Capecitabine,Trastuzumab	6.45	5
**HR-HER2+**	Fam-Trastuzumab Deruxtecan-Nxki	6.45	5

© 2022 Jaber Chehayeb R et al. *JAMA Network Open.*

Subgroup	LineName	Percent (%)	Line Number
**HR-HER2+**	Trastuzumab,Vinorelbine	6.45	5
**HR-HER2+**	Capecitabine,Lapatinib	3.23	5
**HR-HER2+**	Gemcitabine	3.23	5
**HR-HER2+**	Gemcitabine,Trastuzumab	3.23	5
**HR-HER2+**	Ado-Trastuzumab Emtansine,Anastrozole	1.61	5
**HR-HER2+**	Atezolizumab,Paclitaxel Protein-Bound	1.61	5
**HR-HER2+**	Bevacizumab,Capecitabine,Trastuzumab	1.61	5
**HR-HER2+**	Bevacizumab,Doxorubicin Pegylated Liposomal	1.61	5
**HR-HER2+**	Capecitabine	1.61	5
**HR-HER2+**	Capecitabine,Ixabepilone,Lapatinib,Trastuzumab	1.61	5
**HR-HER2+**	Capecitabine,Lapatinib,Trastuzumab	1.61	5
**HR-HER2+**	Carboplatin,Fam-Trastuzumab Deruxtecan-Nxki,Gemcitabine	1.61	5
**HR-HER2+**	Cyclophosphamide,Docetaxel	1.61	5
**HR-HER2+**	Cyclophosphamide,Doxorubicin	1.61	5
**HR-HER2+**	Docetaxel,Trastuzumab	1.61	5
**HR-HER2+**	Doxorubicin	1.61	5
**HR-HER2+**	Doxorubicin Pegylated Liposomal,Lapatinib,Trastuzumab	1.61	5
**HR-HER2+**	Eribulin	1.61	5
**HR-HER2+**	Eribulin,Trastuzumab	1.61	5
**HR-HER2+**	Eribulin,Trastuzumab-Anns	1.61	5
**HR-HER2+**	Fam-Trastuzumab Deruxtecan-Nxki,Trastuzumab	1.61	5
**HR-HER2+**	Fluorouracil,Lapatinib,Methotrexate,Trastuzumab	1.61	5
**HR-HER2+**	Gemcitabine,Margetuximab-Cmkb	1.61	5
**HR-HER2+**	Gemcitabine,Nivolumab	1.61	5
**HR-HER2+**	Gemcitabine,Paclitaxel Protein-Bound,Trastuzumab	1.61	5
**HR-HER2+**	Ixabepilone	1.61	5
**HR-HER2+**	Lapatinib	1.61	5
**HR-HER2+**	Lapatinib,Methotrexate,Trastuzumab	1.61	5
**HR-HER2+**	Lapatinib,Trastuzumab	1.61	5
**HR-HER2+**	Methotrexate	1.61	5
**HR-HER2+**	Methotrexate,Trastuzumab,Vinorelbine	1.61	5
**HR-HER2+**	Neratinib	1.61	5
**HR-HER2+**	Paclitaxel Protein-Bound,Pertuzumab,Trastuzumab	1.61	5
**HR-HER2+**	Paclitaxel Protein-Bound,Trastuzumab	1.61	5
**HR-HER2+**	Pembrolizumab	1.61	5

© 2022 Jaber Chehayeb R et al. *JAMA Network Open.*

Subgroup	LineName	Percent (%)	Line Number
**HR-HER2+**	Trastuzumab	1.61	5
**HR-HER2+**	Trastuzumab-Anns	1.61	5
**HR-HER2+**	Vinorelbine	1.61	5
**HR+HER2+**	Docetaxel,Pertuzumab,Trastuzumab	12.6	1
**HR+HER2+**	Anastrozole	9.34	1
**HR+HER2+**	Letrozole	7.94	1
**HR+HER2+**	Paclitaxel,Pertuzumab,Trastuzumab	5.1	1
**HR+HER2+**	Tamoxifen	5.03	1
**HR+HER2+**	Carboplatin,Docetaxel,Pertuzumab,Trastuzumab	3.84	1
**HR+HER2+**	Letrozole,Palbociclib	3.09	1
**HR+HER2+**	Fulvestrant	2.76	1
**HR+HER2+**	Ado-Trastuzumab Emtansine	2.69	1
**HR+HER2+**	Exemestane	2.26	1
**HR+HER2+**	Fulvestrant,Palbociclib	2.23	1
**HR+HER2+**	Trastuzumab	1.87	1
**HR+HER2+**	Cyclophosphamide,Doxorubicin	1.76	1
**HR+HER2+**	Anastrozole,Trastuzumab	1.44	1
**HR+HER2+**	Letrozole,Trastuzumab	1.4	1
**HR+HER2+**	Capecitabine	1.36	1
**HR+HER2+**	Carboplatin,Docetaxel,Trastuzumab	1.33	1
**HR+HER2+**	Pertuzumab,Trastuzumab	1.29	1
**HR+HER2+**	Capecitabine,Lapatinib	1.11	1
**HR+HER2+**	Paclitaxel,Trastuzumab	1.08	1
**HR+HER2+**	Paclitaxel	1.04	1
**HR+HER2+**	Ado-Trastuzumab Emtansine	8.8	2
**HR+HER2+**	Anastrozole,Trastuzumab	3.74	2
**HR+HER2+**	Anastrozole,Pertuzumab,Trastuzumab	3.59	2
**HR+HER2+**	Letrozole,Pertuzumab,Trastuzumab	3	2
**HR+HER2+**	Docetaxel,Pertuzumab,Trastuzumab	2.9	2
**HR+HER2+**	Fulvestrant	2.85	2
**HR+HER2+**	Letrozole,Trastuzumab	2.56	2
**HR+HER2+**	Capecitabine	2.31	2
**HR+HER2+**	Fulvestrant,Palbociclib	2.11	2
**HR+HER2+**	Letrozole	2.02	2
**HR+HER2+**	Anastrozole	1.97	2

© 2022 Jaber Chehayeb R et al. *JAMA Network Open.*

Subgroup	LineName	Percent (%)	Line Number
**HR+HER2+**	Pertuzumab,Tamoxifen,Trastuzumab	1.97	2
**HR+HER2+**	Exemestane	1.92	2
**HR+HER2+**	Paclitaxel,Pertuzumab,Trastuzumab	1.87	2
**HR+HER2+**	Tamoxifen	1.77	2
**HR+HER2+**	Letrozole,Palbociclib	1.57	2
**HR+HER2+**	Fulvestrant,Trastuzumab	1.47	2
**HR+HER2+**	Capecitabine,Lapatinib	1.38	2
**HR+HER2+**	Exemestane,Trastuzumab	1.38	2
**HR+HER2+**	Tamoxifen,Trastuzumab	1.28	2
**HR+HER2+**	Everolimus,Exemestane	1.23	2
**HR+HER2+**	Paclitaxel	1.13	2
**HR+HER2+**	Ado-Trastuzumab Emtansine	9.61	3
**HR+HER2+**	Capecitabine	3.3	3
**HR+HER2+**	Fulvestrant,Palbociclib	2.7	3
**HR+HER2+**	Docetaxel,Pertuzumab,Trastuzumab	2.48	3
**HR+HER2+**	Letrozole,Pertuzumab,Trastuzumab	2.33	3
**HR+HER2+**	Ado-Trastuzumab Emtansine,Anastrozole	2.25	3
**HR+HER2+**	Fulvestrant	2.1	3
**HR+HER2+**	Capecitabine,Lapatinib	1.95	3
**HR+HER2+**	Anastrozole,Pertuzumab,Trastuzumab	1.8	3
**HR+HER2+**	Eribulin	1.73	3
**HR+HER2+**	Exemestane	1.73	3
**HR+HER2+**	Letrozole,Palbociclib	1.73	3
**HR+HER2+**	Everolimus,Exemestane	1.58	3
**HR+HER2+**	Fulvestrant,Trastuzumab	1.58	3
**HR+HER2+**	Letrozole,Trastuzumab	1.5	3
**HR+HER2+**	Ado-Trastuzumab Emtansine,Letrozole	1.43	3
**HR+HER2+**	Trastuzumab,Vinorelbine	1.43	3
**HR+HER2+**	Letrozole	1.28	3
**HR+HER2+**	Tamoxifen	1.28	3
**HR+HER2+**	Exemestane,Pertuzumab,Trastuzumab	1.2	3
**HR+HER2+**	Fulvestrant,Pertuzumab,Trastuzumab	1.2	3
**HR+HER2+**	Anastrozole	1.13	3
**HR+HER2+**	Paclitaxel,Pertuzumab,Trastuzumab	1.13	3
**HR+HER2+**	Tamoxifen,Trastuzumab	1.13	3

© 2022 Jaber Chehayeb R et al. *JAMA Network Open.*

Subgroup	LineName	Percent (%)	Line Number
**HR+HER2+**	Ado-Trastuzumab Emtansine,Tamoxifen	1.05	3
**HR+HER2+**	Anastrozole,Trastuzumab	1.05	3
**HR+HER2+**	Fam-Trastuzumab Deruxtecan-Nxki	1.05	3
**HR+HER2+**	Ado-Trastuzumab Emtansine	9.06	4
**HR+HER2+**	Capecitabine	3.72	4
**HR+HER2+**	Capecitabine,Lapatinib	3.6	4
**HR+HER2+**	Fulvestrant	2.44	4
**HR+HER2+**	Eribulin	2.32	4
**HR+HER2+**	Fulvestrant,Palbociclib	2.21	4
**HR+HER2+**	Docetaxel,Pertuzumab,Trastuzumab	1.74	4
**HR+HER2+**	Fam-Trastuzumab Deruxtecan-Nxki	1.74	4
**HR+HER2+**	Ado-Trastuzumab Emtansine,Fulvestrant	1.51	4
**HR+HER2+**	Letrozole,Palbociclib	1.51	4
**HR+HER2+**	Trastuzumab,Vinorelbine	1.51	4
**HR+HER2+**	Capecitabine,Trastuzumab	1.39	4
**HR+HER2+**	Ado-Trastuzumab Emtansine,Letrozole	1.28	4
**HR+HER2+**	Anastrozole,Pertuzumab,Trastuzumab	1.28	4
**HR+HER2+**	Paclitaxel Protein-Bound	1.28	4
**HR+HER2+**	Ado-Trastuzumab Emtansine,Anastrozole	1.16	4
**HR+HER2+**	Everolimus,Exemestane	1.16	4
**HR+HER2+**	Fulvestrant,Pertuzumab,Trastuzumab	1.16	4
**HR+HER2+**	Gemcitabine,Trastuzumab	1.16	4
**HR+HER2+**	Tamoxifen	1.16	4
**HR+HER2+**	Anastrozole,Trastuzumab	1.05	4
**HR+HER2+**	Gemcitabine	1.05	4
**HR+HER2+**	Paclitaxel,Pertuzumab,Trastuzumab	1.05	4
**HR+HER2+**	Ado-Trastuzumab Emtansine	8.51	5
**HR+HER2+**	Capecitabine,Lapatinib	3.8	5
**HR+HER2+**	Capecitabine	3.62	5
**HR+HER2+**	Fam-Trastuzumab Deruxtecan-Nxki	3.44	5
**HR+HER2+**	Fulvestrant,Palbociclib	2.54	5
**HR+HER2+**	Everolimus,Exemestane	2.17	5
**HR+HER2+**	Docetaxel,Pertuzumab,Trastuzumab	1.99	5
**HR+HER2+**	Eribulin	1.81	5
**HR+HER2+**	Gemcitabine,Trastuzumab	1.81	5

© 2022 Jaber Chehayeb R et al. *JAMA Network Open.*

Subgroup	LineName	Percent (%)	Line Number
**HR+HER2+**	Capecitabine,Trastuzumab	1.63	5
**HR+HER2+**	Ado-Trastuzumab Emtansine,Exemestane	1.45	5
**HR+HER2+**	Exemestane	1.45	5
**HR+HER2+**	Fulvestrant	1.45	5
**HR+HER2+**	Fulvestrant,Trastuzumab	1.45	5
**HR+HER2+**	Gemcitabine	1.45	5
**HR+HER2+**	Paclitaxel Protein-Bound	1.45	5
**HR+HER2+**	Eribulin,Trastuzumab	1.27	5
**HR+HER2+**	Lapatinib,Trastuzumab	1.09	5
**HR+HER2+**	Paclitaxel	1.09	5
**TNBC**	Capecitabine	19.01	1
**TNBC**	Carboplatin,Gemcitabine	9.13	1
**TNBC**	Cyclophosphamide,Doxorubicin	9.06	1
**TNBC**	Paclitaxel Protein-Bound	7.76	1
**TNBC**	Paclitaxel	7.62	1
**TNBC**	Atezolizumab,Paclitaxel Protein-Bound	5.9	1
**TNBC**	Carboplatin,Paclitaxel	4.8	1
**TNBC**	Eribulin	4.67	1
**TNBC**	Gemcitabine	2.88	1
**TNBC**	Carboplatin,Docetaxel	2.06	1
**TNBC**	Docetaxel	1.85	1
**TNBC**	Carboplatin,Paclitaxel Protein-Bound	1.65	1
**TNBC**	Cyclophosphamide,Docetaxel	1.51	1
**TNBC**	Carboplatin	1.3	1
**TNBC**	Doxorubicin Pegylated Liposomal	1.24	1
**TNBC**	Cyclophosphamide,Docetaxel,Doxorubicin	1.17	1
**TNBC**	Capecitabine,Ixabepilone	1.03	1
**TNBC**	Capecitabine	13.94	2
**TNBC**	Eribulin	10.06	2
**TNBC**	Paclitaxel	9.16	2
**TNBC**	Carboplatin,Gemcitabine	6.32	2
**TNBC**	Paclitaxel Protein-Bound	4.9	2
**TNBC**	Gemcitabine	4.65	2
**TNBC**	Doxorubicin Pegylated Liposomal	4.39	2
**TNBC**	Atezolizumab,Paclitaxel Protein-Bound	4.13	2

© 2022 Jaber Chehayeb R et al. *JAMA Network Open.*

Subgroup	LineName	Percent (%)	Line Number
**TNBC**	Carboplatin,Paclitaxel	3.87	2
**TNBC**	Cyclophosphamide,Doxorubicin	3.74	2
**TNBC**	Vinorelbine	2.45	2
**TNBC**	Capecitabine,Ixabepilone	2.19	2
**TNBC**	Carboplatin	2.19	2
**TNBC**	Ixabepilone	2.06	2
**TNBC**	Docetaxel	1.55	2
**TNBC**	Olaparib	1.29	2
**TNBC**	Carboplatin,Paclitaxel Protein-Bound	1.03	2
**TNBC**	Eribulin	18.53	3
**TNBC**	Capecitabine	14.72	3
**TNBC**	Gemcitabine	8.12	3
**TNBC**	Carboplatin,Gemcitabine	5.33	3
**TNBC**	Atezolizumab,Paclitaxel Protein-Bound	4.57	3
**TNBC**	Doxorubicin Pegylated Liposomal	4.57	3
**TNBC**	Paclitaxel Protein-Bound	3.81	3
**TNBC**	Sacituzumab Govitecan-Hziy	3.05	3
**TNBC**	Paclitaxel	2.79	3
**TNBC**	Ixabepilone	2.54	3
**TNBC**	Vinorelbine	2.54	3
**TNBC**	Carboplatin	2.03	3
**TNBC**	Capecitabine,Ixabepilone	1.52	3
**TNBC**	Cyclophosphamide,Doxorubicin	1.52	3
**TNBC**	Nivolumab	1.27	3
**TNBC**	Olaparib	1.27	3
**TNBC**	Carboplatin,Paclitaxel	1.02	3
**TNBC**	Carboplatin,Paclitaxel Protein-Bound	1.02	3
**TNBC**	Cisplatin	1.02	3
**TNBC**	Doxorubicin	1.02	3
**TNBC**	Eribulin	14.65	4
**TNBC**	Gemcitabine	9.6	4
**TNBC**	Capecitabine	8.59	4
**TNBC**	Vinorelbine	8.08	4
**TNBC**	Doxorubicin Pegylated Liposomal	7.07	4
**TNBC**	Paclitaxel Protein-Bound	6.06	4

© 2022 Jaber Chehayeb R et al. *JAMA Network Open*.

Subgroup	LineName	Percent (%)	Line Number
**TNBC**	Carboplatin,Gemcitabine	4.55	4
**TNBC**	Ixabepilone	4.55	4
**TNBC**	Atezolizumab,Paclitaxel Protein-Bound	3.03	4
**TNBC**	Pembrolizumab	3.03	4
**TNBC**	Sacituzumab Govitecan-Hziy	3.03	4
**TNBC**	Carboplatin,Paclitaxel Protein-Bound	1.52	4
**TNBC**	Nivolumab	1.52	4
**TNBC**	Olaparib	1.52	4
**TNBC**	Capecitabine,Gemcitabine	1.01	4
**TNBC**	Carboplatin	1.01	4
**TNBC**	Cyclophosphamide,Docetaxel	1.01	4
**TNBC**	Doxorubicin	1.01	4
**TNBC**	Eribulin	10.98	5
**TNBC**	Vinorelbine	10.98	5
**TNBC**	Carboplatin,Gemcitabine	8.54	5
**TNBC**	Paclitaxel Protein-Bound	8.54	5
**TNBC**	Doxorubicin Pegylated Liposomal	7.32	5
**TNBC**	Capecitabine	4.88	5
**TNBC**	Carboplatin	4.88	5
**TNBC**	Sacituzumab Govitecan-Hziy	4.88	5
**TNBC**	Atezolizumab,Paclitaxel Protein-Bound	3.66	5
**TNBC**	Gemcitabine	3.66	5
**TNBC**	Ixabepilone	3.66	5
**TNBC**	Cyclophosphamide,Fluorouracil,Methotrexate	2.44	5
**TNBC**	Docetaxel	2.44	5
**TNBC**	Pembrolizumab	2.44	5
**TNBC**	Cyclophosphamide,Methotrexate	1.22	5
**TNBC**	Temozolomide	1.22	5
**TNBC**	Atezolizumab,Capecitabine,Paclitaxel Protein-Bound,Tucatinib	1.22	5
**TNBC**	Capecitabine,Gemcitabine,Vinorelbine	1.22	5
**TNBC**	Capecitabine,Ixabepilone	1.22	5
**TNBC**	Capecitabine,Lapatinib	1.22	5
**TNBC**	Carboplatin,Eribulin,Gemcitabine	1.22	5
**TNBC**	Carboplatin,Paclitaxel Protein-Bound	1.22	5
**TNBC**	Carboplatin,Vinorelbine	1.22	5

Subgroup	LineName	Percent (%)	Line Number
**TNBC**	Cisplatin,Olaparib	1.22	5
**TNBC**	Cyclophosphamide,Docetaxel,Doxorubicin	1.22	5
**TNBC**	Doxorubicin Pegylated Liposomal,Everolimus	1.22	5
**TNBC**	Everolimus	1.22	5
**TNBC**	Everolimus,Ixabepilone	1.22	5
**TNBC**	Gemcitabine,Paclitaxel Protein-Bound	1.22	5
**TNBC**	Nivolumab	1.22	5
**TNBC**	Nivolumab,Olaparib,Paclitaxel Protein-Bound	1.22	5

© 2022 Jaber Chehayeb R et al. *JAMA Network Open.*

eTable 4. Most Frequently Used Drugs at a Yearly Level by Patient Count Per Drug for Each Subgroup and Year

Subgroup	Drug	Route	Year	Patient Count
HR+HER2-	palbociclib	Oral	1	3362
HR+HER2-	letrozole	Oral	1	3357
HR+HER2-	fulvestrant	Intramuscular	1	3138
HR+HER2-	anastrozole	Oral	1	1706
HR+HER2-	capecitabine	Oral	1	1078
HR+HER2-	fulvestrant	Intramuscular	2	2325
HR+HER2-	palbociclib	Oral	2	1513
HR+HER2-	letrozole	Oral	2	1326
HR+HER2-	capecitabine	Oral	2	793
HR+HER2-	exemestane	Oral	2	791
HR+HER2-	fulvestrant	Intramuscular	3	1561
HR+HER2-	palbociclib	Oral	3	1028
HR+HER2-	letrozole	Oral	3	790
HR+HER2-	capecitabine	Oral	3	545
HR+HER2-	exemestane	Oral	3	540
HR+HER2-	fulvestrant	Intramuscular	4	983
HR+HER2-	palbociclib	Oral	4	701
HR+HER2-	letrozole	Oral	4	513
HR+HER2-	capecitabine	Oral	4	345
HR+HER2-	exemestane	Oral	4	323
HR+HER2-	fulvestrant	Intramuscular	5	622
HR+HER2-	palbociclib	Oral	5	436
HR+HER2-	letrozole	Oral	5	307
HR+HER2-	capecitabine	Oral	5	206
HR+HER2-	exemestane	Oral	5	160
HR+HER2-	fulvestrant	Intramuscular	6	365
HR+HER2-	palbociclib	Oral	6	235
HR+HER2-	letrozole	Oral	6	142
HR+HER2-	capecitabine	Oral	6	124
HR+HER2-	exemestane	Oral	6	88
HR+HER2-	fulvestrant	Intramuscular	7	212
HR+HER2-	palbociclib	Oral	7	129
HR+HER2-	letrozole	Oral	7	81
HR+HER2-	capecitabine	Oral	7	64
HR+HER2-	exemestane	Oral	7	59
HR+HER2-	fulvestrant	Intramuscular	8	116

© 2022 Jaber Chehayeb R et al. *JAMA Network Open.*

Subgroup	Drug	Route	Year	Patient Count
HR+HER2-	palbociclib	Oral	8	59
HR+HER2-	letrozole	Oral	8	42
HR+HER2-	capecitabine	Oral	8	28
HR+HER2-	exemestane	Oral	8	24
HR+HER2-	fulvestrant	Intramuscular	9	51
HR+HER2-	palbociclib	Oral	9	27
HR+HER2-	letrozole	Oral	9	23
HR+HER2-	capecitabine	Oral	9	14
HR+HER2-	anastrozole	Oral	9	13
HR+HER2-	fulvestrant	Intramuscular	10	16
HR+HER2-	palbociclib	Oral	10	12
HR+HER2-	letrozole	Oral	10	11
HR+HER2-	anastrozole	Oral	10	9
HR+HER2-	exemestane	Oral	10	7
HR+HER2+	trastuzumab	Intravenous	1	1595
HR+HER2+	pertuzumab	Intravenous	1	1206
HR+HER2+	docetaxel	Intravenous	1	870
HR+HER2+	letrozole	Oral	1	573
HR+HER2+	anastrozole	Oral	1	455
HR+HER2+	trastuzumab	Intravenous	2	1001
HR+HER2+	pertuzumab	Intravenous	2	667
HR+HER2+	ado-trastuzumab emtansine	Intravenous	2	378
HR+HER2+	fulvestrant	Intramuscular	2	347
HR+HER2+	letrozole	Oral	2	287
HR+HER2+	trastuzumab	Intravenous	3	609
HR+HER2+	pertuzumab	Intravenous	3	373
HR+HER2+	ado-trastuzumab emtansine	Intravenous	3	284
HR+HER2+	fulvestrant	Intramuscular	3	250
HR+HER2+	letrozole	Oral	3	172
HR+HER2+	trastuzumab	Intravenous	4	386
HR+HER2+	pertuzumab	Intravenous	4	208
HR+HER2+	ado-trastuzumab emtansine	Intravenous	4	184
HR+HER2+	fulvestrant	Intramuscular	4	171
HR+HER2+	letrozole	Oral	4	109
HR+HER2+	trastuzumab	Intravenous	5	224
HR+HER2+	pertuzumab	Intravenous	5	130
HR+HER2+	fulvestrant	Intramuscular	5	108

© 2022 Jaber Chehayeb R et al. *JAMA Network Open.*

Subgroup	Drug	Route	Year	Patient Count
HR+HER2+	ado-trastuzumab emtansine	Intravenous	5	104
HR+HER2+	letrozole	Oral	5	64
HR+HER2+	trastuzumab	Intravenous	6	130
HR+HER2+	pertuzumab	Intravenous	6	80
HR+HER2+	ado-trastuzumab emtansine	Intravenous	6	59
HR+HER2+	fulvestrant	Intramuscular	6	59
HR+HER2+	trastuzumab-anns	Intravenous	6	44
HR+HER2+	trastuzumab	Intravenous	7	80
HR+HER2+	pertuzumab	Intravenous	7	49
HR+HER2+	fulvestrant	Intramuscular	7	35
HR+HER2+	trastuzumab-anns	Intravenous	7	33
HR+HER2+	ado-trastuzumab emtansine	Intravenous	7	32
HR+HER2+	trastuzumab	Intravenous	8	39
HR+HER2+	trastuzumab-anns	Intravenous	8	21
HR+HER2+	ado-trastuzumab emtansine	Intravenous	8	17
HR+HER2+	pertuzumab	Intravenous	8	17
HR+HER2+	letrozole	Oral	8	16
HR+HER2+	trastuzumab	Intravenous	9	16
HR+HER2+	trastuzumab-anns	Intravenous	9	14
HR+HER2+	ado-trastuzumab emtansine	Intravenous	9	8
HR+HER2+	fulvestrant	Intramuscular	9	8
HR+HER2+	pertuzumab	Intravenous	9	8
HR+HER2+	trastuzumab	Intravenous	10	5
HR+HER2+	letrozole	Oral	10	4
HR+HER2+	ado-trastuzumab emtansine	Intravenous	10	4
HR+HER2+	fulvestrant	Intramuscular	10	4
HR+HER2+	palbociclib	Oral	10	3
HR-HER2+	trastuzumab	Intravenous	1	566
HR-HER2+	pertuzumab	Intravenous	1	415
HR-HER2+	docetaxel	Intravenous	1	331
HR-HER2+	paclitaxel	Intravenous	1	173
HR-HER2+	ado-trastuzumab emtansine	Intravenous	1	144
HR-HER2+	trastuzumab	Intravenous	2	292
HR-HER2+	pertuzumab	Intravenous	2	188
HR-HER2+	ado-trastuzumab emtansine	Intravenous	2	125
HR-HER2+	capecitabine	Oral	2	83
HR-HER2+	lapatinib	Oral	2	58

© 2022 Jaber Chehayeb R et al. *JAMA Network Open.*

Subgroup	Drug	Route	Year	Patient Count
HR-HER2+	trastuzumab	Intravenous	3	156
HR-HER2+	pertuzumab	Intravenous	3	105
HR-HER2+	ado-trastuzumab emtansine	Intravenous	3	85
HR-HER2+	capecitabine	Oral	3	43
HR-HER2+	lapatinib	Oral	3	38
HR-HER2+	trastuzumab	Intravenous	4	87
HR-HER2+	pertuzumab	Intravenous	4	53
HR-HER2+	ado-trastuzumab emtansine	Intravenous	4	43
HR-HER2+	capecitabine	Oral	4	22
HR-HER2+	lapatinib	Oral	4	20
HR-HER2+	trastuzumab	Intravenous	5	60
HR-HER2+	pertuzumab	Intravenous	5	40
HR-HER2+	ado-trastuzumab emtansine	Intravenous	5	23
HR-HER2+	trastuzumab-anns	Intravenous	5	12
HR-HER2+	lapatinib	Oral	5	7
HR-HER2+	trastuzumab	Intravenous	6	37
HR-HER2+	pertuzumab	Intravenous	6	25
HR-HER2+	ado-trastuzumab emtansine	Intravenous	6	16
HR-HER2+	trastuzumab-anns	Intravenous	6	10
HR-HER2+	capecitabine	Oral	6	5
HR-HER2+	trastuzumab	Intravenous	7	20
HR-HER2+	ado-trastuzumab emtansine	Intravenous	7	13
HR-HER2+	pertuzumab	Intravenous	7	13
HR-HER2+	trastuzumab-anns	Intravenous	7	8
HR-HER2+	fam-trastuzumab deruxtecan-nxki	Intravenous	7	3
HR-HER2+	trastuzumab	Intravenous	8	10
HR-HER2+	ado-trastuzumab emtansine	Intravenous	8	6
HR-HER2+	trastuzumab-anns	Intravenous	8	6
HR-HER2+	pertuzumab	Intravenous	8	4
HR-HER2+	lapatinib	Oral	8	1
HR-HER2+	trastuzumab	Intravenous	9	8
HR-HER2+	trastuzumab-anns	Intravenous	9	5
HR-HER2+	pertuzumab	Intravenous	9	4
HR-HER2+	ado-trastuzumab emtansine	Intravenous	9	2
HR-HER2+	lapatinib	Oral	9	1
HR-HER2+	trastuzumab	Intravenous	10	3
HR-HER2+	trastuzumab-anns	Intravenous	10	3

© 2022 Jaber Chehayeb R et al. *JAMA Network Open.*

Subgroup	Drug	Route	Year	Patient Count
HR-HER2+	ado-trastuzumab emtansine	Intravenous	10	2
HR-HER2+	lapatinib	Oral	10	1
HR-HER2+	pertuzumab	Intravenous	10	1
TNBC	capecitabine	Oral	1	448
TNBC	carboplatin	Intravenous	1	399
TNBC	paclitaxel protein-bound	Intravenous	1	370
TNBC	gemcitabine	Intravenous	1	352
TNBC	paclitaxel	Intravenous	1	341
TNBC	capecitabine	Oral	2	136
TNBC	gemcitabine	Intravenous	2	105
TNBC	paclitaxel protein-bound	Intravenous	2	104
TNBC	eribulin	Intravenous	2	89
TNBC	carboplatin	Intravenous	2	72
TNBC	capecitabine	Oral	3	42
TNBC	paclitaxel protein-bound	Intravenous	3	36
TNBC	gemcitabine	Intravenous	3	23
TNBC	eribulin	Intravenous	3	23
TNBC	doxorubicin pegylated liposomal	Intravenous	3	17
TNBC	paclitaxel protein-bound	Intravenous	4	11
TNBC	capecitabine	Oral	4	9
TNBC	gemcitabine	Intravenous	4	8
TNBC	vinorelbine	Intravenous	4	8
TNBC	eribulin	Intravenous	4	8
TNBC	paclitaxel protein-bound	Intravenous	5	9
TNBC	capecitabine	Oral	5	4
TNBC	atezolizumab	Intravenous	5	4
TNBC	doxorubicin pegylated liposomal	Intravenous	5	3
TNBC	eribulin	Intravenous	5	3
TNBC	capecitabine	Oral	6	2
TNBC	olaparib	Oral	6	2
TNBC	doxorubicin pegylated liposomal	Intravenous	6	2
TNBC	nivolumab	Intravenous	6	2
TNBC	anastrozole	Oral	6	1
TNBC	capecitabine	Oral	7	1
TNBC	olaparib	Oral	7	1
TNBC	doxorubicin pegylated liposomal	Intravenous	7	1
TNBC	carboplatin	Intravenous	7	1

© 2022 Jaber Chehayeb R et al. *JAMA Network Open.*

Subgroup	Drug	Route	Year	Patient Count
TNBC	gemcitabine	Intravenous	7	1
TNBC	letrozole	Oral	8	1
TNBC	doxorubicin pegylated liposomal	Intravenous	8	1

© 2022 Jaber Chehayeb R et al. *JAMA Network Open.*

eTable 5. Percentage of Patients Receiving Supportive Drugs by Subgroup and Line Number

Line Number	Subgroup	# of Patients Receiving Supportive Drugs	Total # of patients	% of patients
1	HR+HER2-	1,434	10,171	14.1
1	HR+HER2+	854	2,785	30.7
1	HR-HER2+	361	802	45.0
1	TNBC	671	1457	46.1
2	HR+HER2-	1,031	6,546	15.8
2	HR+HER2+	376	2,034	18.5
2	HR-HER2+	112	407	27.5
2	TNBC	351	775	45.3
3	HR+HER2-	763	4,179	18.3
3	HR+HER2+	234	1,132	20.7
3	HR-HER2+	67	220	30.5
3	TNBC	163	394	41.4
4	HR+HER2-	627	2,640	23.8
4	HR+HER2+	160	861	18.6
4	HR-HER2+	33	113	29.2
4	TNBC	87	198	43.9
5	HR+HER2-	510	1,676	30.4
5	HR-HER2+	22	62	35.5
5	HR+HER2+	124	552	22.5
5	TNBC	33	82	40.2

© 2022 Jaber Chehayeb R et al. *JAMA Network Open.*

eTable 6. Annual AWP Anticancer and Supportive Drug Costs by Year After Diagnosis and Subgroup

*Note the listed cost for all years for anti-cancer, supportive and total cost represents the mean cumulative cost per patient by subgroup; this cost is less than the sum of Years 1 through 10 since not all patients had ten years of treatment data.

	Year	HR+HER2-	HR+HER2+	HR-HER2+	TNBC
	1	762 87	183,726	163,977	41,838
	2	71,082	166,042	159,327	49,485
	3	71,876	152,540	160,251	65,672
	4	75,955	153,012	160,409	82,495
	5	71,621	150,048	150,021	117,169
	6	72,856	144,384	168,687	85,204
	7	68,567	137,424	139,538	48,518
	8	67,755	122,509	117,956	22,451
	9	58,607	93,911	99,388	
	10	58,365	113,053	48,301	
	**All Years**	171,243	455,722	374,166	66,466
	1	8,718	21,412	28,390	25,161
	2	7,374	7,010	9,428	20,575
	3	7,073	6,334	6,106	22,531
	4	8,444	6,199	4,681	10,372
	5	7,555	6,047	6,543	7,988
	6	6,904	4,981	5,889	2,723
	7	5,267	4,713	2,053	3,655
	8	5,058	5,747	2,221	
	9	3,690	6,404		
	10	2,605			
**Supportive Cost**	**All Years**	18,381	32,348	36,969	33,496
**Total Cost**	**All Years**	189,624	488,069	411,135	99,962

© 2022 Jaber Chehayeb R et al. *JAMA Network Open.*

eTable 7. AWP and Medicare Anticancer and Supportive Drug Costs by Line and Subgroup for the First 5 Lines of Therapy

Subgroup	Line#	MC Anti-cancer cost	AWP Anti-cancer cost	MC Supportive cost	AWP Supportive cost	Total MC cost	Total AWP cost	Patient Count
**HR+HER2-**	1	68,389	106,107	831	2,670	69,220	108,777	5,346
**HR+HER2-**	2	42,762	77,238	617	1,889	43,379	79,127	3,958
**HR+HER2-**	3	31,038	59,459	853	2,740	31,891	62,199	2,512
**HR+HER2-**	4	26,857	51,000	1,434	4,555	28,291	55,555	1,566
**HR+HER2-**	5	22,323	41,521	1,619	5,050	23,942	46,572	1,052
**HR-HER2+**	1	170,017	229,339	4,119	13,528	174,136	242,867	619
**HR-HER2+**	2	103,500	140,931	956	2,917	104,455	143,848	291
**HR-HER2+**	3	73,702	100,003	1,605	5,198	75,307	105,202	174
**HR-HER2+**	4	62,113	86,687	1,650	5,020	63,763	91,707	107
**HR-HER2+**	5	46,227	63,594	1,591	5,325	47,818	68,919	58
**HR+HER2+**	1	104,072	143,341	3,542	11,733	107,614	155,073	1,448
**HR+HER2+**	2	112,508	155,217	686	2,190	113,194	157,407	1,062
**HR+HER2+**	3	95,752	130,148	590	1,988	96,342	132,136	696
**HR+HER2+**	4	78,320	110,305	970	3,045	79,290	113,350	435
**HR+HER2+**	5	56,467	82,434	707	2,227	57,173	84,661	260
**TNBC**	1	15,633	26,515	2,879	9,256	18,512	35,771	1,169
**TNBC**	2	18,804	28,535	2,449	7,396	21,253	35,932	599
**TNBC**	3	20,775	29,595	2,228	6,994	23,002	36,588	319
**TNBC**	4	18,841	27,510	1,976	6,279	20,817	33,789	159
**TNBC**	5	21,750	29,367	1,876	6,234	23,627	35,601	80

*MC=Medicare

© 2022 Jaber Chehayeb R et al. *JAMA Network Open.*

eTable 8. Most Costly Drug Regimens by Subgroup and Associated Costs for Each of the First 5 Lines of Therapy

**Line Name**	**Sub group**	**Line Number**	**AWP price**	**Medicare Price**
Letrozole,Palbociclib	HR+HER 2-	1	296,254	222,014
Letrozole,Ribociclib	HR+HER 2-	1	253,055	62,503
Fulvestrant,Palbociclib	HR+HER 2-	1	239,214	160,297
Anastrozole,Palbociclib	HR+HER 2-	1	200,210	151,987
Everolimus,Exemestane	HR+HER 2-	1	197,012	69,085
Letrozole,Ribociclib	HR+HER 2-	2	264,010	65,693
Letrozole,Palbociclib	HR+HER 2-	2	222,493	168,132
Fulvestrant,Palbociclib	HR+HER 2-	2	165,117	105,901
Abemaciclib,Fulvestrant	HR+HER 2-	2	155,189	107,218
Anastrozole,Palbociclib	HR+HER 2-	2	154,057	118,046
Letrozole,Palbociclib	HR+HER 2-	3	181,994	136,788
Fulvestrant,Palbociclib	HR+HER 2-	3	140,394	91,193
Abemaciclib,Fulvestrant	HR+HER 2-	3	137,184	94,408
Alpelisib,Fulvestrant	HR+HER 2-	3	115,001	45,194
Everolimus,Exemestane	HR+HER 2-	3	111,387	39,068
Fulvestrant,Palbociclib	HR+HER 2-	4	140,857	91,393
Abemaciclib,Fulvestrant	HR+HER 2-	4	130,635	93,287
Letrozole,Palbociclib	HR+HER 2-	4	126,064	94,798
Everolimus,Exemestane	HR+HER 2-	4	121,555	42,651
Alpelisib,Fulvestrant	HR+HER 2-	4	82,708	32,672
Fulvestrant,Letrozole,Palbociclib	HR+HER 2-	5	154,818	106,372
Abemaciclib,Fulvestrant	HR+HER 2-	5	137,461	93,246
Everolimus,Exemestane	HR+HER 2-	5	131,404	46,122

© 2022 Jaber Chehayeb R et al. *JAMA Network Open.*

Line Name	Sub group	Line Number	AWP price	Medicare Price
Alpelisib,Fulvestrant	HR+HER 2-	5	120,526	47,694
Letrozole,Palbociclib	HR+HER 2-	5	114,412	85,434
Docetaxel,Paclitaxel,Pertuzumab,Trastuzumab	HR+HER 2+	1	234,848	178,587
Docetaxel,Pertuzumab,Trastuzumab	HR+HER 2+	1	224,545	168,540
Letrozole,Palbociclib	HR+HER 2+	1	219,389	164,079
Pertuzumab,Trastuzumab	HR+HER 2+	1	205,594	160,568
Paclitaxel,Pertuzumab,Trastuzumab	HR+HER 2+	1	195,807	150,807
Docetaxel,Letrozole,Pertuzumab,Trastuzumab	HR+HER 2+	2	420,572	313,396
Letrozole,Pertuzumab,Trastuzumab	HR+HER 2+	2	347,565	264,705
Anastrozole,Pertuzumab,Trastuzumab	HR+HER 2+	2	295,560	226,217
Fulvestrant,Pertuzumab,Trastuzumab	HR+HER 2+	2	279,621	193,815
Anastrozole,Docetaxel,Pertuzumab,Trastuzumab	HR+HER 2+	2	265,590	197,326
Pertuzumab,Tamoxifen,Trastuzumab	HR+HER 2+	3	427,251	330,625
Letrozole,Pertuzumab,Trastuzumab	HR+HER 2+	3	270,049	206,946
Fulvestrant,Pertuzumab,Trastuzumab	HR+HER 2+	3	248,925	175,370
Exemestane,Trastuzumab	HR+HER 2+	3	220,762	157,962
Anastrozole,Pertuzumab,Trastuzumab	HR+HER 2+	3	213,129	165,406
Exemestane,Pertuzumab,Trastuzumab	HR+HER 2+	4	414,320	319,237
Anastrozole,Pertuzumab,Trastuzumab	HR+HER 2+	4	300,214	231,188
Ado-Trastuzumab Emtansine,Tamoxifen	HR+HER 2+	4	246,748	212,900
Paclitaxel,Pertuzumab,Trastuzumab	HR+HER 2+	4	232,821	184,162
Capecitabine,Trastuzumab	HR+HER 2+	4	197,832	90,525
Fulvestrant,Trastuzumab	HR+HER 2+	5	186,164	110,470
Anastrozole,Pertuzumab,Trastuzumab	HR+HER 2+	5	173,217	134,913

© 2022 Jaber Chehayeb R et al. *JAMA Network Open.*

Line Name	Sub group	Line Number	AWP price	Medicare Price
Docetaxel,Pertuzumab,Trastuzumab	HR+HER 2+	5	169,705	123,636
Paclitaxel Protein-Bound,Trastuzumab	HR+HER 2+	5	160,436	118,311
Ado-Trastuzumab Emtansine,Fulvestrant	HR+HER 2+	5	158,601	121,836
Docetaxel,Paclitaxel,Pertuzumab,Trastuzumab	HR-HER2+	1	389,615	295,333
Docetaxel,Pertuzumab,Trastuzumab	HR-HER2+	1	381,274	289,128
Carboplatin,Docetaxel,Pertuzumab,Trastuzumab	HR-HER2+	1	345,698	260,457
Paclitaxel Protein-Bound,Pertuzumab,Trastuzumab	HR-HER2+	1	320,343	248,619
Paclitaxel,Pertuzumab,Trastuzumab-Anns	HR-HER2+	1	298,780	205,220
Paclitaxel,Pertuzumab,Trastuzumab	HR-HER2+	2	367,037	281,797
Docetaxel,Trastuzumab	HR-HER2+	2	316,569	213,453
Lapatinib,Trastuzumab	HR-HER2+	2	306,330	161,069
Pertuzumab,Trastuzumab,Vinorelbine	HR-HER2+	2	301,908	237,066
Docetaxel,Pertuzumab,Trastuzumab	HR-HER2+	2	265,094	201,847
Pertuzumab,Trastuzumab,Vinorelbine	HR-HER2+	3	547,755	417,915
Capecitabine,Pertuzumab,Trastuzumab	HR-HER2+	3	339,783	242,442
Capecitabine,Trastuzumab-Anns,Tucatinib	HR-HER2+	3	171,079	125,233
Trastuzumab	HR-HER2+	3	164,891	117,569
Ado-Trastuzumab Emtansine,Neratinib	HR-HER2+	3	164,289	107,679
Alpelisib,Paclitaxel Protein-Bound	HR-HER2+	4	318,322	172,880
Paclitaxel,Pertuzumab,Trastuzumab	HR-HER2+	4	316,858	254,198
Capecitabine,Trastuzumab,Tucatinib	HR-HER2+	4	282,427	212,008
Capecitabine,Trastuzumab-Anns,Tucatinib	HR-HER2+	4	267,288	191,819
Eribulin,Lapatinib	HR-HER2+	4	251,596	208,202
Carboplatin,Fam-Trastuzumab Deruxtecan-Nxki,Gemcitabine	HR-HER2+	5	212,997	182,040

© 2022 Jaber Chehayeb R et al. *JAMA Network Open.*

Line Name	Sub group	Line Number	AWP price	Medicare Price
Fam-Trastuzumab Deruxtecan-Nxki	HR-HER2+	5	159,662	136,501
Fam-Trastuzumab Deruxtecan-Nxki,Trastuzumab	HR-HER2+	5	144,227	117,266
Paclitaxel Protein-Bound,Pertuzumab,Trastuzumab	HR-HER2+	5	136,751	107,321
Ado-Trastuzumab Emtansine	HR-HER2+	5	124,605	107,679
Atezolizumab,Paclitaxel Protein-Bound	TNBC	1	130,678	106,683
Capecitabine,Ixabepilone	TNBC	1	62,689	42,115
Paclitaxel Protein-Bound	TNBC	1	52,120	40,437
Carboplatin,Paclitaxel Protein-Bound	TNBC	1	43,903	33,758
Eribulin	TNBC	1	34,687	28,704
Atezolizumab,Paclitaxel Protein-Bound	TNBC	2	160,657	131,055
Sacituzumab Govitecan-Hziy	TNBC	2	142,744	121,762
Carboplatin,Paclitaxel Protein-Bound	TNBC	2	78,241	60,391
Capecitabine,Ixabepilone	TNBC	2	73,774	49,558
Olaparib	TNBC	2	73,617	32,442
Sacituzumab Govitecan-Hziy	TNBC	3	178,278	152,073
Atezolizumab,Paclitaxel Protein-Bound	TNBC	3	78,697	64,162
Capecitabine,Sacituzumab Govitecan-Hziy	TNBC	3	68,322	58,279
Capecitabine,Ixabepilone	TNBC	3	45,462	28,864
Pembrolizumab	TNBC	3	36,242	31,652
Olaparib	TNBC	4	137,265	60,491
Atezolizumab,Paclitaxel Protein-Bound	TNBC	4	114,184	93,071
Sacituzumab Govitecan-Hziy	TNBC	4	108,888	92,883
Ixabepilone	TNBC	4	54,366	45,967
Pembrolizumab	TNBC	4	34,229	29,893
Atezolizumab,Paclitaxel Protein-Bound	TNBC	5	174,032	142,229
Sacituzumab Govitecan-Hziy	TNBC	5	112,091	95,615
Pembrolizumab	TNBC	5	78,525	68,580
Doxorubicin Pegylated Liposomal,Everolimus	TNBC	5	71,447	28,967
Carboplatin,Paclitaxel Protein-Bound	TNBC	5	59,278	45,430

© 2022 Jaber Chehayeb R et al. *JAMA Network Open.*

eTable 9. Mean and Median Medicare Costs for Patients Receiving Supportive Care by Subgroup and Year

**Subgroup**	**Y**	**Mean (2021 USD)**	**SD (2021 USD)**	**Median (2021 USD)**	**Q1**	**Q3**	**# Receiving Supportive Drugs**	**Total #**	**%**
HR+HER2-	1	14,025	13,996	9,367	2,819	21,295	2,033	9,581	21
HR+HER2-	2	13,507	16,170	7,754	1,910	18,491	956	4,929	19
HR+HER2-	3	13,087	15,596	7,044	2,347	18,718	644	3,272	20
HR+HER2-	4	14,981	15,973	9,101	3,522	22,216	416	2,039	20
HR+HER2-	5	14,310	17,991	6,928	2,115	19,999	240	1,278	19
HR+HER2-	6	13,646	14,725	8,887	2,091	19,167	133	745	18
HR+HER2-	7	11,567	16,460	4,815	1,824	13,377	70	443	16
HR+HER2-	8	9,765	9,746	7,357	3,114	13,008	43	226	19
HR+HER2-	9	8,217	11,212	4,437	2,150	9,507	18	118	15
HR+HER2-	10	9,017	7,924	8,887	3,463	14,441	4	48	8
**HR+HER2-**	**All**	**20,668**	**28,375**	**11,744**	**3,522**	**26,660**	**3,036**	**9,767**	**31**
HR+HER2+	1	17,250	14,318	15,208	4,443	26,660	1,075	2,652	41
HR+HER2+	2	13,389	17,172	7,007	2,115	17,773	322	1,740	19
HR+HER2+	3	12,407	16,265	5,854	1,410	17,773	224	1,208	19
HR+HER2+	4	12,790	15,578	7,790	2,362	17,735	138	755	18
HR+HER2+	5	12,622	14,591	8,897	1,702	16,196	82	463	18
HR+HER2+	6	11,113	15,533	4,691	1,539	13,596	47	296	16
HR+HER2+	7	10,610	13,645	4,735	1,410	10,629	27	178	15
HR+HER2+	8	14,997	13,895	5,639	4,443	31,103	13	97	13
HR+HER2+	9	15,136	15,066	10,952	2,574	24,638	7	55	13
**HR+HER2+**	**All**	**23,136**	**28,602**	**17,773**	**4,735**	**28,821**	**1,278**	**2,703**	**47**
HR-HER2+	1	17,675	14,747	14,720	4,443	26,660	392	760	52
HR-HER2+	2	14,961	19,268	7,287	1,326	19,943	94	427	22

© 2022 Jaber Chehayeb R et al. *JAMA Network Open.*

Subgroup	Y	Mean (2021 USD)	SD (2021 USD)	Median (2021 USD)	Q1	Q3	# Receiving Supportive Drugs	Total #	%
HR-HER2+	3	11,189	13,888	6,459	1,434	16,526	56	266	21
HR-HER2+	4	8,517	15,146	4,206	1,410	8,956	32	158	20
HR-HER2+	5	21,904	25,341	15,341	4,397	23,390	9	96	9
HR-HER2+	6	15,326	25,483	5,285	1,410	13,813	7	57	12
HR-HER2+	7	5,544	8,322	1,922	480	4,934	5	39	13
HR-HER2+	8	5,134	1,197	4,443	4,443	5,480	3	24	13
**HR-HER2+**	**All**	**22,179**	**30,347**	**16,079**	**4,480**	**28,630**	**432**	**787**	**55**
TNBC	1	15,433	14,515	11,706	4,229	23,061	787	1,410	56
TNBC	2	14,299	16,505	8,887	2,534	17,773	201	407	49
TNBC	3	17,226	21,160	6,827	2,146	24,670	57	126	45
TNBC	4	9,499	9,277	6,191	1,310	15,476	19	49	39
TNBC	5	9,662	10,897	4,934	3,248	12,714	7	20	35
TNBC	6	3,121	3,417	2,101	628	4,594	4	8	50
TNBC	7	7,280		7,280	7,280	7,280	1	4	25
**TNBC**	**All**	**19,392**	**23,616**	**13,036**	**4,443**	**26,411**	**840**	**1,425**	**59**

© 2022 Jaber Chehayeb R et al. *JAMA Network Open*.

eTable 10. Annual Median Medicare Anticancer Drug Costs for Patients by Year After Diagnosis and Subgroup

Subgroup	Year	Median(USD))	Q1(USD)	Q3(USD)
HR+HER2-	1	13,179	959	88,364
HR+HER2-	2	13,006	2,379	63,396
HR+HER2-	3	16,088	2,876	64,673
HR+HER2-	4	19,361	3,515	68,073
HR+HER2-	5	15,805	3,239	62,356
HR+HER2-	6	16,178	2,605	64,055
HR+HER2-	7	8,690	1,598	57,775
HR+HER2-	8	9,879	1,985	60,158
HR+HER2-	9	6,173	323	51,980
HR+HER2-	10	5,391	265	48,652
**HR+HER2-**	**All**	**53,686**	**3,261**	**168,262**
HER2+HR+	1	107,239	20,510	185,388
HER2+HR+	2	93,098	29,611	175,689
HER2+HR+	3	87,537	24,316	171,492
HER2+HR+	4	92,954	31,195	165,448
HER2+HR+	5	92,954	29,834	161,938
HER2+HR+	6	90,684	22,246	169,596
HER2+HR+	7	91,281	30,871	152,734
HER2+HR+	8	71,786	22,777	123,062
HER2+HR+	9	52,125	7,298	103,674
HER2+HR+	10	83,269	12,146	135,369
**HR+HER2+**	**All**	**160,764**	**51,890**	**390,239**
HR-HER2+	1	111,341	40,553	173,950
HR-HER2+	2	109,624	51,370	176,346
HR-HER2+	3	117,240	39,841	174,338
HR-HER2+	4	103,167	43,785	184,363
HR-HER2+	5	95,821	55,297	167,653
HR-HER2+	6	123,298	92,954	184,363
HR-HER2+	7	92,954	76,812	140,473
HR-HER2+	8	85,331	36,879	106,130
HR-HER2+	9	61,296	29,448	99,814
HR-HER2+	10	20,474	12,517	22,587
**HR-HER2+**	**All**	**203,789**	**51,386**	**450,590**
TNBC	1	3,240	689	30,749
TNBC	2	12,549	929	42,327
TNBC	3	20,975	1,528	58,817

© 2022 Jaber Chehayeb R et al. *JAMA Network Open.*

**Subgroup**	**Year**	**Median(USD))**	**Q1(USD)**	**Q3(USD)**
TNBC	4	18,590	371	49,019
TNBC	5	10,744	6,574	85,191
TNBC	6	26,473	710	66,522
TNBC	7	23,497	14,277	31,157
TNBC	8	10,548	5,291	15,805
**TNBC**	**All**	**8,963**	**831**	**44,873**

© 2022 Jaber Chehayeb R et al. *JAMA Network Open.*

eTable 11. Drugs That Made up the 3 Largest Contributors to Overall Yearly Medicare Costs Stratified by Subgroup and Year After Diagnosis

Subgroup	Drug Name	Route	Year	Patient Count	Total Medicare Cost	Total AWP Cost
HR+HER2-	palbociclib	Oral	1	3362	311,752,981	402,355,045
HR+HER2-	abemaciclib	Oral	1	434	32,734,606	40,066,241
HR+HER2-	paclitaxel protein-bound	IV	1	828	30,127,595	38,832,217
HR+HER2-	palbociclib	Oral	2	1513	107,526,084	138,775,458
HR+HER2-	abemaciclib	Oral	2	234	15,192,756	18,595,508
HR+HER2-	everolimus	Oral	2	526	13,966,908	39,682,949
HR+HER2-	palbociclib	Oral	3	1028	69,541,052	89,751,166
HR+HER2-	everolimus	Oral	3	382	10,724,895	30,471,702
HR+HER2-	abemaciclib	Oral	3	149	9,611,258	11,763,911
HR+HER2-	palbociclib	Oral	4	701	48,551,598	62,661,727
HR+HER2-	everolimus	Oral	4	224	6,130,542	17,418,171
HR+HER2-	eribulin	IV	4	168	5,201,342	6,285,427
HR+HER2-	palbociclib	Oral	5	436	29,081,388	37,533,059
HR+HER2-	eribulin	IV	5	108	3,401,879	4,110,912
HR+HER2-	abemaciclib	Oral	5	80	3,254,807	3,983,792
HR+HER2-	palbociclib	Oral	6	235	14,830,870	19,141,037
HR+HER2-	abemaciclib	Oral	6	59	3,404,311	4,166,782
HR+HER2-	everolimus	Oral	6	73	2,177,058	6,185,483
HR+HER2-	palbociclib	Oral	7	129	9,186,223	11,855,936
HR+HER2-	abemaciclib	Oral	7	34	1,754,276	2,147,185
HR+HER2-	everolimus	Oral	7	36	901,954	2,562,644
HR+HER2-	palbociclib	Oral	8	59	3,885,795	5,015,089
HR+HER2-	abemaciclib	Oral	8	17	950,519	1,163,408
HR+HER2-	alpelisib	Oral	8	20	635,239	1,463,766
HR+HER2-	palbociclib	Oral	9	27	1,668,999	2,154,046
HR+HER2-	abemaciclib	Oral	9	10	869,137	1,063,800
HR+HER2-	alpelisib	Oral	9	6	312,738	720,635
HR+HER2-	palbociclib	Oral	10	12	1,113,390	1,436,964
HR+HER2-	alpelisib	Oral	10	5	182,892	421,433
HR+HER2-	everolimus	Oral	10	2	86,713	246,369
HR-HER2+	trastuzumab	IV	1	566	44,562,871	61,861,038
HR-HER2+	pertuzumab	IV	1	415	27,370,770	31,900,117
HR-HER2+	ado-trastuzumab emtansine	IV	1	144	9,445,039	10,929,682
HR-HER2+	trastuzumab	IV	2	292	20,220,947	28,070,201
HR-HER2+	pertuzumab	IV	2	188	13,414,923	15,634,840
HR-HER2+	ado-trastuzumab emtansine	IV	2	125	9,424,529	10,905,948
HR-HER2+	trastuzumab	IV	3	156	11,957,437	16,599,008

© 2022 Jaber Chehayeb R et al. *JAMA Network Open.*

Subgroup	Drug Name	Route	Year	Patient Count	Total Medicare Cost	Total AWP Cost
HR-HER2+	pertuzumab	IV	3	105	7,772,228	9,058,386
HR-HER2+	ado-trastuzumab emtansine	IV	3	85	6,799,198	7,867,947
HR-HER2+	trastuzumab	IV	4	87	7,428,268	10,311,731
HR-HER2+	pertuzumab	IV	4	53	4,543,764	5,295,672
HR-HER2+	ado-trastuzumab emtansine	IV	4	43	4,225,142	4,889,282
HR-HER2+	trastuzumab	IV	5	60	4,693,523	6,515,428
HR-HER2+	pertuzumab	IV	5	40	2,932,379	3,417,633
HR-HER2+	ado-trastuzumab emtansine	IV	5	23	2,061,295	2,385,305
HR-HER2+	trastuzumab	IV	6	37	2,680,858	3,721,499
HR-HER2+	pertuzumab	IV	6	25	2,135,228	2,488,568
HR-HER2+	ado-trastuzumab emtansine	IV	6	16	1,753,639	2,029,289
HR-HER2+	trastuzumab	IV	7	20	1,423,953	1,976,696
HR-HER2+	ado-trastuzumab emtansine	IV	7	13	1,199,858	1,388,461
HR-HER2+	pertuzumab	IV	7	13	990,746	1,154,695
HR-HER2+	trastuzumab	IV	8	10	792,133	1,099,619
HR-HER2+	ado-trastuzumab emtansine	IV	8	6	553,781	640,828
HR-HER2+	pertuzumab	IV	8	4	381,494	444,624
HR-HER2+	trastuzumab	IV	9	8	432,440	600,302
HR-HER2+	ado-trastuzumab emtansine	IV	9	2	276,890	320,414
HR-HER2+	pertuzumab	IV	9	4	261,921	305,264
HR-HER2+	trastuzumab	IV	10	3	57,928	80,414
HR-HER2+	trastuzumab-anns	IV	10	3	43,876	89,764
HR-HER2+	ado-trastuzumab emtansine	IV	10	2	20,510	23,734
HR+HER2+	trastuzumab	IV	1	1595	167,413,542	232,399,197
HR+HER2+	pertuzumab	IV	1	1206	107,216,890	124,959,269
HR+HER2+	palbociclib	Oral	1	280	22,634,580	29,212,672
HR+HER2+	trastuzumab	IV	2	1001	89,338,594	124,017,552
HR+HER2+	pertuzumab	IV	2	667	53,335,136	62,161,098
HR+HER2+	ado-trastuzumab emtansine	IV	2	378	27,760,826	32,124,483
HR+HER2+	trastuzumab	IV	3	609	53,541,726	74,325,254
HR+HER2+	pertuzumab	IV	3	373	29,853,327	34,793,492
HR+HER2+	ado-trastuzumab emtansine	IV	3	284	22,007,659	25,466,990
HR+HER2+	trastuzumab	IV	4	386	31,205,730	43,318,996
HR+HER2+	pertuzumab	IV	4	208	16,711,714	19,477,188
HR+HER2+	ado-trastuzumab emtansine	IV	4	184	16,192,961	18,738,293
HR+HER2+	trastuzumab	IV	5	224	16,929,823	23,501,547
HR+HER2+	pertuzumab	IV	5	130	9,326,674	10,870,063
HR+HER2+	ado-trastuzumab emtansine	IV	5	104	8,870,748	10,265,119
HR+HER2+	trastuzumab	IV	6	130	10,828,512	15,031,864

© 2022 Jaber Chehayeb R et al. *JAMA Network Open.*

Subgroup	Drug Name	Route	Year	Patient Count	Total Medicare Cost	Total AWP Cost
HR+HER2+	pertuzumab	IV	6	80	6,001,413	6,994,534
HR+HER2+	ado-trastuzumab emtansine	IV	6	59	5,906,995	6,835,501
HR+HER2+	trastuzumab	IV	7	80	6,136,337	8,518,306
HR+HER2+	ado-trastuzumab emtansine	IV	7	32	3,404,726	3,939,907
HR+HER2+	pertuzumab	IV	7	49	3,131,667	3,649,899
HR+HER2+	ado-trastuzumab emtansine	IV	8	17	2,409,972	2,788,790
HR+HER2+	trastuzumab	IV	8	39	2,366,969	3,285,766
HR+HER2+	pertuzumab	IV	8	17	1,360,852	1,586,047
HR+HER2+	trastuzumab	IV	9	16	835,242	1,159,462
HR+HER2+	ado-trastuzumab emtansine	IV	9	8	758,885	878,172
HR+HER2+	pertuzumab	IV	9	8	449,821	524,258
HR+HER2+	ado-trastuzumab emtansine	IV	10	4	399,953	462,820
HR+HER2+	palbociclib	Oral	10	3	378,075	487,952
HR+HER2+	pertuzumab	IV	10	3	153,736	179,177
TNBC	paclitaxel protein-bound	IV	1	370	12,477,853	16,083,019
TNBC	atezolizumab	IV	1	141	8,018,602	9,521,740
TNBC	eribulin	IV	1	206	4,967,114	6,002,381
TNBC	paclitaxel protein-bound	IV	2	104	2,944,908	3,795,767
TNBC	sacituzumab govitecan-hziy	IV	2	22	2,622,586	3,074,496
TNBC	atezolizumab	IV	2	39	2,331,223	2,768,225
TNBC	sacituzumab govitecan-hziy	IV	3	13	1,420,567	1,665,352
TNBC	paclitaxel protein-bound	IV	3	36	1,233,145	1,589,432
TNBC	eribulin	IV	3	23	684,094	826,675
TNBC	paclitaxel protein-bound	IV	4	11	777,050	1,001,560
TNBC	nivolumab	IV	4	4	720,420	846,062
TNBC	atezolizumab	IV	4	6	710,085	843,195
TNBC	nivolumab	IV	5	2	630,368	740,304
TNBC	paclitaxel protein-bound	IV	5	9	270,278	348,369
TNBC	atezolizumab	IV	5	4	247,860	294,323
TNBC	nivolumab	IV	6	2	187,032	219,651
TNBC	olaparib	Oral	6	2	103,408	234,652
TNBC	sacituzumab govitecan-hziy	IV	6	1	54,637	64,052
TNBC	olaparib	Oral	7	1	28,272	64,154
TNBC	paclitaxel protein-bound	IV	7	1	28,154	36,288
TNBC	doxorubicin pegylated liposomal	IV	7	1	18,722	38,720
TNBC	doxorubicin pegylated liposomal	IV	8	1	21,063	43,560
TNBC	letrozole	Oral	8	1	33	1,342

© 2022 Jaber Chehayeb R et al. *JAMA Network Open.*

eTable 12. Lines That Made up the 3 Largest Contributors to Medicare Costs by Calendar Year Stratified by Subgroup

Year	Sub group	Line Name	Mean Medicare Price	Mean AWP price
2011	HR+HER2-	Paclitaxel Protein-Bound	31,157	40,159
2011	HR+HER2-	Eribulin	20,448	24,710
2011	HR+HER2-	Fulvestrant	6,465	47,073
2011	HR-HER2+	Eribulin,Lapatinib	208,202	251,597
2011	HR-HER2+	Carboplatin,Docetaxel,Trastuzumab	123,958	179,022
2011	HR-HER2+	Paclitaxel,Trastuzumab	98,897	138,221
2011	HR+HER2+	Exemestane,Trastuzumab	215,765	307,483
2011	HR+HER2+	Letrozole,Trastuzumab	205,278	287,287
2011	HR+HER2+	Fulvestrant,Trastuzumab	156,917	245,449
2011	TNBC	Paclitaxel Protein-Bound	45,046	58,061
2011	TNBC	Capecitabine,Ixabepilone	30,365	39,766
2011	TNBC	Eribulin	29,743	35,942
2012	HR+HER2-	Eribulin	47,802	57,765
2012	HR+HER2-	Everolimus,Exemestane	47,206	134,555
2012	HR+HER2-	Paclitaxel Protein-Bound	36,427	46,952

© 2022 Jaber Chehayeb R et al. *JAMA Network Open.*

Year	Sub group	Line Name	Mean Medicare Price	Mean AWP price
2012	HR-HER2+	Pertuzumab,Trastuzumab,Vinorelbine	545,173	694,510
2012	HR-HER2+	Paclitaxel,Pertuzumab,Trastuzumab	440,974	589,099
2012	HR-HER2+	Docetaxel,Pertuzumab,Trastuzumab	428,990	569,737
2012	HR+HER2+	Docetaxel,Paclitaxel,Pertuzumab,Trastuzumab	434,550	558,304
2012	HR+HER2+	Letrozole,Pertuzumab,Trastuzumab	422,640	537,941
2012	HR+HER2+	Docetaxel,Pertuzumab,Trastuzumab	314,292	419,709
2012	TNBC	Paclitaxel,Paclitaxel Protein-Bound	59,915	78,639
2012	TNBC	Capecitabine,Ixabepilone	49,257	78,302
2012	TNBC	Carboplatin,Paclitaxel Protein-Bound	33,987	44,189
2013	HR+HER2-	Everolimus,Exemestane	68,629	195,585
2013	HR+HER2-	Ixabepilone	50,452	59,670
2013	HR+HER2-	Paclitaxel Protein-Bound	35,087	45,224
2013	HR-HER2+	Paclitaxel,Pertuzumab,Trastuzumab	410,009	523,221
2013	HR-HER2+	Docetaxel,Paclitaxel,Pertuzumab,Trastuzumab	370,305	485,686
2013	HR-HER2+	Docetaxel,Pertuzumab,Trastuzumab	314,649	414,364
2013	HR+HER2+	Ado-Trastuzumab Emtansine,Anastrozole	1,066,541	1,234,188

© 2022 Jaber Chehayeb R et al. *JAMA Network Open.*

Year	Sub group	Line Name	Mean Medicare Price	Mean AWP price
2013	HR+HER2+	Letrozole,Pertuzumab,Trastuzumab	771,481	1,011,517
2013	HR+HER2+	Ado-Trastuzumab Emtansine,Tamoxifen	358,932	415,352
2013	TNBC	Paclitaxel Protein-Bound	56,091	72,298
2013	TNBC	Carboplatin,Paclitaxel Protein-Bound	51,123	66,734
2013	TNBC	Capecitabine,Ixabepilone	44,157	70,005
2014	HR+HER2-	Letrozole,Palbociclib	358,259	476,732
2014	HR+HER2-	Ixabepilone	100,904	119,340
2014	HR+HER2-	Everolimus,Exemestane	55,579	158,362
2014	HR-HER2+	Carboplatin,Docetaxel,Pertuzumab,Trastuzumab	502,893	650,544
2014	HR-HER2+	Docetaxel,Pertuzumab,Trastuzumab	399,835	525,215
2014	HR-HER2+	Paclitaxel Protein-Bound,Pertuzumab,Trastuzumab	354,506	461,727
2014	HR+HER2+	Docetaxel,Letrozole,Pertuzumab,Trastuzumab	432,433	594,426
2014	HR+HER2+	Pertuzumab,Trastuzumab	383,721	494,738
2014	HR+HER2+	Pertuzumab,Tamoxifen,Trastuzumab	314,967	409,193
2014	TNBC	Capecitabine,Ixabepilone	36,805	52,884
2014	TNBC	Carboplatin,Paclitaxel Protein-Bound	32,594	42,419

© 2022 Jaber Chehayeb R et al. *JAMA Network Open.*

Year	Sub group	Line Name	Mean Medicare Price	Mean AWP price
2014	TNBC	Paclitaxel Protein-Bound	32,429	41,799
2015	HR+HER2-	Anastrozole,Palbociclib	264,873	341,964
2015	HR+HER2-	Letrozole,Palbociclib	210,672	280,432
2015	HR+HER2-	Fulvestrant,Palbociclib	163,205	246,201
2015	HR-HER2+	Capecitabine,Pertuzumab,Trastuzumab	411,709	523,339
2015	HR-HER2+	Docetaxel,Pertuzumab,Trastuzumab	288,608	379,625
2015	HR-HER2+	Carboplatin,Docetaxel,Pertuzumab,Trastuzumab	211,403	285,098
2015	HR+HER2+	Docetaxel,Letrozole,Pertuzumab,Trastuzumab	629,583	831,800
2015	HR+HER2+	Ado-Trastuzumab Emtansine,Letrozole	548,653	634,895
2015	HR+HER2+	Pertuzumab,Tamoxifen,Trastuzumab	389,718	497,386
2015	TNBC	Carboplatin,Paclitaxel Protein-Bound	78,098	101,050
2015	TNBC	Paclitaxel,Paclitaxel Protein-Bound	68,001	88,419
2015	TNBC	Nivolumab	48,490	56,946
2016	HR+HER2-	Letrozole,Palbociclib	239,605	319,690
2016	HR+HER2-	Fulvestrant,Palbociclib	144,915	218,649
2016	HR+HER2-	Anastrozole,Palbociclib	144,472	188,814

© 2022 Jaber Chehayeb R et al. *JAMA Network Open.*

Year	Sub group	Line Name	Mean Medicare Price	Mean AWP price
2016	HR-HER2+	Docetaxel,Paclitaxel,Pertuzumab,Trastuzumab	561,644	721,835
2016	HR-HER2+	Docetaxel,Pertuzumab,Trastuzumab	277,144	366,130
2016	HR-HER2+	Capecitabine,Pertuzumab,Trastuzumab	253,166	416,923
2016	HR+HER2+	Docetaxel,Letrozole,Pertuzumab,Trastuzumab	378,275	510,077
2016	HR+HER2+	Letrozole,Pertuzumab,Trastuzumab	342,007	445,316
2016	HR+HER2+	Pertuzumab,Tamoxifen,Trastuzumab	311,924	399,708
2016	TNBC	Capecitabine,Ixabepilone	65,872	89,620
2016	TNBC	Ixabepilone	41,803	49,441
2016	TNBC	Olaparib	34,894	79,181
2017	HR+HER2-	Letrozole,Palbociclib	211,639	282,397
2017	HR+HER2-	Anastrozole,Palbociclib	188,821	248,107
2017	HR+HER2-	Fulvestrant,Palbociclib	138,888	212,153
2017	HR-HER2+	Pertuzumab,Trastuzumab,Vinorelbine	349,281	451,556
2017	HR-HER2+	Paclitaxel Protein-Bound,Pertuzumab,Trastuzumab	319,479	411,491
2017	HR-HER2+	Docetaxel,Paclitaxel,Pertuzumab,Trastuzumab	314,571	437,197
2017	HR+HER2+	Letrozole,Pertuzumab,Trastuzumab	354,785	466,080

© 2022 Jaber Chehayeb R et al. *JAMA Network Open.*

Year	Sub group	Line Name	Mean Medicare Price	Mean AWP price
2017	HR+HER2+	Anastrozole,Docetaxel,Pertuzumab,Trastuzumab	327,820	442,163
2017	HR+HER2+	Docetaxel,Paclitaxel,Pertuzumab,Trastuzumab	275,844	366,050
2017	TNBC	Capecitabine,Ixabepilone	50,936	80,169
2017	TNBC	Carboplatin,Paclitaxel Protein-Bound	49,116	63,901
2017	TNBC	Paclitaxel Protein-Bound	47,080	60,682
2018	HR+HER2-	Letrozole,Palbociclib	213,247	283,797
2018	HR+HER2-	Anastrozole,Palbociclib	164,683	216,731
2018	HR+HER2-	Abemaciclib,Fulvestrant	126,813	182,099
2018	HR-HER2+	Docetaxel,Pertuzumab,Trastuzumab-Anns	419,972	595,294
2018	HR-HER2+	Carboplatin,Docetaxel,Pertuzumab,Trastuzumab	234,090	317,829
2018	HR-HER2+	Ixabepilone	211,899	250,614
2018	HR+HER2+	Exemestane,Pertuzumab,Trastuzumab	264,641	344,067
2018	HR+HER2+	Anastrozole,Pertuzumab,Trastuzumab	223,155	291,696
2018	HR+HER2+	Fulvestrant,Pertuzumab,Trastuzumab	210,446	297,769
2018	TNBC	Pembrolizumab	52,754	60,404
2018	TNBC	Ixabepilone	45,407	53,703

© 2022 Jaber Chehayeb R et al. *JAMA Network Open.*

Year	Sub group	Line Name	Mean Medicare Price	Mean AWP price
2018	TNBC	Atezolizumab,Paclitaxel Protein-Bound	43,688	53,592
2019	HR+HER2-	Letrozole,Palbociclib	164,099	218,146
2019	HR+HER2-	Anastrozole,Palbociclib	155,259	204,567
2019	HR+HER2-	Abemaciclib,Fulvestrant	141,452	201,117
2019	HR-HER2+	Paclitaxel,Pertuzumab,Trastuzumab-Anns	372,235	518,503
2019	HR-HER2+	Docetaxel,Pertuzumab,Trastuzumab-Anns	218,006	329,683
2019	HR-HER2+	Paclitaxel Protein-Bound,Pertuzumab,Trastuzumab	195,856	250,927
2019	HR+HER2+	Docetaxel,Letrozole,Pertuzumab,Trastuzumab	217,177	284,371
2019	HR+HER2+	Docetaxel,Pertuzumab,Trastuzumab-Anns	177,295	257,371
2019	HR+HER2+	Exemestane,Pertuzumab,Trastuzumab	158,969	205,530
2019	TNBC	Atezolizumab,Paclitaxel Protein-Bound	116,310	142,506
2019	TNBC	Nivolumab,Olaparib,Paclitaxel Protein-Bound	41,785	50,685
2019	TNBC	Paclitaxel Protein-Bound	41,605	53,626
2020	HR+HER2-	Letrozole,Palbociclib	100,259	133,358
2020	HR+HER2-	Anastrozole,Palbociclib	90,021	118,205
2020	HR+HER2-	Abemaciclib,Fulvestrant	70,506	102,399

© 2022 Jaber Chehayeb R et al. *JAMA Network Open*.

Year	Sub group	Line Name	Mean Medicare Price	Mean AWP price
2020	HR-HER2+	Capecitabine,Trastuzumab,Tucatinib	212,009	282,427
2020	HR-HER2+	Capecitabine,Trastuzumab-Anns,Tucatinib	178,206	244,258
2020	HR-HER2+	Alpelisib,Paclitaxel Protein-Bound	172,880	318,323
2020	HR+HER2+	Pertuzumab,Trastuzumab	173,280	220,255
2020	HR+HER2+	Letrozole,Pertuzumab,Trastuzumab	164,676	218,399
2020	HR+HER2+	Ado-Trastuzumab Emtansine,Exemestane	143,999	167,530
2020	TNBC	Sacituzumab Govitecan-Hziy	154,805	181,481
2020	TNBC	Atezolizumab,Paclitaxel Protein-Bound	100,892	123,626
2020	TNBC	Capecitabine,Sacituzumab Govitecan-Hziy	71,028	83,268

© 2022 Jaber Chehayeb R et al. *JAMA Network Open.*

eTable 13. Mean Medicare and AWP Costs by Year After Diagnosis Stratified by Year of Diagnosis

			Societal Perspective (Medicare)			Payor Perspective (AWP)		
**SG**	**Y**	**YoD**	**Anti -Cancer**	**Supportive**	**Total**	**Anti-Cancer**	**Supportive**	**Total**
HR+HER2-	1	2011	8,889	4,097	12,986	23,661	12,248	35,909
HR+HER2-	1	2012	9,105	4,350	13,455	26,490	12,933	39,423
HR+HER2-	1	2013	10,601	3,902	14,503	28,548	11,443	39,990
HR+HER2-	1	2014	13,404	2,881	16,284	32,139	8,472	40,611
HR+HER2-	1	2015	39,875	3,459	43,334	64,266	9,941	74,207
HR+HER2-	1	2016	54,894	2,823	57,716	84,521	8,440	92,961
HR+HER2-	1	2017	62,073	2,953	65,026	97,417	9,044	106,461
HR+HER2-	1	2018	71,137	2,154	73,292	111,352	6,359	117,710
HR+HER2-	1	2019	78,112	2,451	80,563	120,926	6,756	127,682
HR+HER2-	2	2011	17,687	4,481	22,168	42,754	12,766	55,520
HR+HER2-	2	2012	15,966	3,894	19,861	42,918	10,942	53,860
HR+HER2-	2	2013	18,122	3,411	21,534	45,147	8,994	54,141
HR+HER2-	2	2014	31,640	2,578	34,218	62,348	7,822	70,170
HR+HER2-	2	2015	39,924	3,734	43,658	68,877	10,274	79,151
HR+HER2-	2	2016	45,157	2,645	47,802	78,574	7,647	86,221
HR+HER2-	2	2017	48,753	2,216	50,969	86,057	6,461	92,518
HR+HER2-	2	2018	50,724	1,765	52,489	89,628	4,710	94,338
HR+HER2-	3	2011	17,861	3,279	21,140	45,440	9,136	54,576
HR+HER2-	3	2012	20,142	4,304	24,446	52,581	12,836	65,417
HR+HER2-	3	2013	33,344	3,207	36,551	64,818	8,054	72,872
HR+HER2-	3	2014	36,521	2,436	38,957	68,994	7,116	76,109
HR+HER2-	3	2015	41,068	3,228	44,296	74,197	9,123	83,320
HR+HER2-	3	2016	43,430	3,303	46,733	77,913	8,559	86,472
HR+HER2-	3	2017	51,150	2,486	53,636	93,622	6,955	100,576
HR+HER2-	4	2011	18,266	3,673	21,939	48,207	10,672	58,878
HR+HER2-	4	2012	32,104	4,420	36,524	64,377	13,370	77,747
HR+HER2-	4	2013	43,041	5,515	48,556	78,486	14,922	93,408
HR+HER2-	4	2014	40,236	3,233	43,470	76,092	9,551	85,643
HR+HER2-	4	2015	44,569	2,524	47,093	81,285	6,592	87,877
HR+HER2-	4	2017	56,643	1,844	58,487	97,190	4,969	102,159
HR+HER2-	5	2011	31,946	5,108	37,054	62,253	14,077	76,330
HR+HER2-	5	2012	36,731	2,155	38,886	69,491	6,512	76,003
HR+HER2-	5	2013	37,029	4,882	41,911	70,426	13,690	84,115
HR+HER2-	5	2014	42,717	2,430	45,148	77,393	7,417	84,809

© 2022 Jaber Chehayeb R et al. *JAMA Network Open.*

			Societal Perspective (Medicare)			Payor Perspective (AWP)		
**SG**	**Y**	**YoD**	**Anti -Cancer**	**Supportive**	**Total**	**Anti-Cancer**	**Supportive**	**Total**
HR+HER2-	5	2017	18,196	633	18,830	30,380	1,571	31,950
HR-HER2+	1	2011	121,890	10,060	131,950	185,065	31,056	216,120
HR-HER2+	1	2012	108,071	7,907	115,977	163,173	25,868	189,041
HR-HER2+	1	2013	116,586	7,499	124,084	164,681	23,997	188,678
HR-HER2+	1	2014	124,860	10,844	135,703	165,962	36,090	202,052
HR-HER2+	1	2015	116,273	10,080	126,353	156,072	28,622	184,694
HR-HER2+	1	2016	119,194	8,448	127,642	159,203	26,979	186,183
HR-HER2+	1	2017	121,404	9,871	131,275	163,517	30,866	194,383
HR-HER2+	1	2018	125,899	9,685	135,584	172,905	32,846	205,752
HR-HER2+	1	2019	108,612	6,839	115,451	152,937	20,839	173,776
HR-HER2+	2	2011	84,433	5,994	90,427	137,201	16,073	153,275
HR-HER2+	2	2012	91,753	2,374	94,127	129,770	6,851	136,621
HR-HER2+	2	2013	123,718	2,598	126,317	168,413	8,326	176,739
HR-HER2+	2	2014	144,344	4,722	149,066	193,598	14,348	207,947
HR-HER2+	2	2015	121,235	1,946	123,181	159,833	4,662	164,495
HR-HER2+	2	2016	131,992	2,719	134,711	172,881	7,488	180,370
HR-HER2+	2	2017	110,570	3,474	114,045	153,621	10,176	163,797
HR-HER2+	2	2018	124,754	3,957	128,711	174,801	12,747	187,548
HR-HER2+	3	2011	74,759	2,551	77,310	100,900	6,957	107,857
HR-HER2+	3	2012	122,564	2,734	125,297	164,497	6,982	171,479
HR-HER2+	3	2013	117,359	2,369	119,728	158,434	6,977	165,412
HR-HER2+	3	2014	166,348	3,920	170,268	218,380	11,863	230,243
HR-HER2+	3	2015	112,990	1,754	114,744	160,938	4,535	165,473
HR-HER2+	3	2016	129,355	1,902	131,257	174,767	6,354	181,121
HR-HER2+	3	2017	108,294	3,338	111,632	155,782	5,706	161,487
HR-HER2+	4	2011	101,939	3,843	105,783	141,286	11,434	152,719
HR-HER2+	4	2012	91,852	533	92,385	122,451	653	123,104
HR-HER2+	4	2013	124,239	1,268	125,507	172,981	3,371	176,352
HR-HER2+	4	2014	163,429	5,983	169,413	213,456	19,312	232,767
HR-HER2+	4	2015	114,058	254	114,312	165,095	447	165,542
HR-HER2+	4	2017	140,982	1,142	142,124	192,724	1,577	194,302
HR-HER2+	5	2011	44,994	5,005	49,999	63,475	17,101	80,576
HR-HER2+	5	2012	126,190	0	126,190	174,066	0	174,066
HR-HER2+	5	2013	110,645	0	110,645	154,705	0	154,705
HR-HER2+	5	2014	168,496	7,292	175,788	219,627	22,939	242,566
HR-HER2+	5	2017	38,313	0	38,313	51,440	0	51,440

© 2022 Jaber Chehayeb R et al. *JAMA Network Open.*

			Societal Perspective (Medicare)			Payor Perspective (AWP)		
**SG**	**Y**	**YoD**	**Anti -Cancer**	**Supportive**	**Total**	**Anti-Cancer**	**Supportive**	**Total**
HR+HER2+	1	2011	94,967	5,030	99,997	147,527	15,625	163,152
HR+HER2+	1	2012	102,269	6,549	108,819	151,482	20,115	171,597
HR+HER2+	1	2013	118,964	8,980	127,944	166,927	27,527	194,454
HR+HER2+	1	2014	117,711	6,017	123,728	164,122	18,565	182,687
HR+HER2+	1	2015	129,105	7,854	136,959	178,511	23,842	202,353
HR+HER2+	1	2016	133,800	6,223	140,024	184,554	19,897	204,451
HR+HER2+	1	2017	152,001	7,490	159,491	207,108	23,018	230,127
HR+HER2+	1	2018	148,827	7,355	156,182	203,528	22,567	226,095
HR+HER2+	1	2019	150,193	6,520	156,712	208,920	19,437	228,357
HR+HER2+	2	2011	88,291	3,000	91,291	138,440	9,470	147,911
HR+HER2+	2	2012	99,274	2,884	102,158	144,497	7,527	152,024
HR+HER2+	2	2013	116,895	2,895	119,790	162,344	9,164	171,508
HR+HER2+	2	2014	114,549	3,333	117,882	160,534	9,825	170,359
HR+HER2+	2	2015	130,446	2,021	132,467	183,067	5,890	188,957
HR+HER2+	2	2016	133,207	2,856	136,063	185,737	8,708	194,445
HR+HER2+	2	2017	125,710	1,903	127,613	174,835	4,823	179,658
HR+HER2+	2	2018	128,019	3,014	131,033	184,113	7,562	191,676
HR+HER2+	3	2011	91,467	2,244	93,711	135,458	6,262	141,720
HR+HER2+	3	2012	97,242	2,402	99,644	145,159	5,225	150,384
HR+HER2+	3	2013	108,986	2,904	111,890	152,819	8,433	161,252
HR+HER2+	3	2014	112,172	4,042	116,214	156,092	12,412	168,504
HR+HER2+	3	2015	127,262	2,343	129,605	177,727	6,106	183,833
HR+HER2+	3	2016	129,455	1,583	131,038	182,901	4,342	187,244
HR+HER2+	3	2017	114,951	1,757	116,708	163,838	4,456	168,294
HR+HER2+	4	2011	97,932	1,314	99,246	138,823	2,956	141,779
HR+HER2+	4	2012	92,723	2,173	94,896	131,080	4,461	135,541
HR+HER2+	4	2013	104,154	3,955	108,108	145,986	12,533	158,519
HR+HER2+	4	2014	127,028	3,049	130,077	177,405	8,459	185,864
HR+HER2+	4	2015	138,731	2,840	141,570	195,246	6,582	201,828
HR+HER2+	4	2017	103,054	1,179	104,233	143,340	2,812	146,152
HR+HER2+	5	2011	90,562	3,189	93,751	129,862	9,318	139,180
HR+HER2+	5	2012	122,434	4,691	127,125	172,304	13,670	185,974
HR+HER2+	5	2013	101,527	3,217	104,745	140,201	8,978	149,179
HR+HER2+	5	2014	120,788	1,940	122,728	174,877	4,980	179,857
HR+HER2+	5	2017	35,847	627	36,474	48,835	973	49,809
TNBC	1	2011	21,389	10,007	31,397	36,713	31,684	68,397

© 2022 Jaber Chehayeb R et al. *JAMA Network Open.*

			Societal Perspective (Medicare)			Payor Perspective (AWP)		
**SG**	**Y**	**YoD**	**Anti -Cancer**	**Supportive**	**Total**	**Anti-Cancer**	**Supportive**	**Total**
TNBC	1	2012	14,372	9,193	23,565	29,297	26,444	55,742
TNBC	1	2013	10,398	9,989	20,387	22,110	27,686	49,796
TNBC	1	2014	21,303	11,722	33,024	35,419	32,489	67,908
TNBC	1	2015	19,507	9,606	29,114	33,654	30,016	63,670
TNBC	1	2016	18,104	7,808	25,912	35,706	23,348	59,054
TNBC	1	2017	17,018	8,370	25,388	30,448	24,998	55,446
TNBC	1	2018	24,917	7,817	32,733	40,765	23,754	64,519
TNBC	1	2019	46,752	7,022	53,775	65,831	20,073	85,904
TNBC	2	2011	26,096	3,526	29,621	45,268	11,114	56,382
TNBC	2	2012	22,134	6,475	28,609	32,958	17,162	50,119
TNBC	2	2013	13,611	8,841	22,452	25,613	23,600	49,213
TNBC	2	2014	33,657	9,809	43,466	47,806	28,784	76,590
TNBC	2	2015	16,322	6,040	22,362	30,385	19,064	49,449
TNBC	2	2016	13,782	7,551	21,333	26,555	21,851	48,406
TNBC	2	2017	25,617	4,004	29,620	39,356	11,327	50,683
TNBC	2	2018	57,777	8,656	66,433	77,471	24,981	102,452
TNBC	3	2011	33,121	1,143	34,264	58,176	4,079	62,255
TNBC	3	2012	14,129	6,368	20,497	26,549	17,103	43,652
TNBC	3	2013	21,258	3,047	24,304	37,901	5,579	43,480
TNBC	3	2014	45,775	9,364	55,139	64,107	26,338	90,444
TNBC	3	2015	14,549	3,012	17,561	40,194	8,072	48,266
TNBC	3	2016	20,074	10,080	30,155	35,988	30,909	66,897
TNBC	3	2017	30,571	8,422	38,993	60,465	25,596	86,061
TNBC	4	2011	44,942	857	45,799	59,855	2,061	61,916
TNBC	4	2012	15,484	7,252	22,735	21,328	23,893	45,221
TNBC	4	2013	45,741	16,784	62,526	61,693	35,085	96,777
TNBC	4	2014	52,395	1,195	53,590	67,836	2,728	70,564
TNBC	4	2015	15,664	630	16,295	28,108	1,823	29,931
TNBC	4	2017	283,781	6,152	289,933	364,420	14,397	378,817
TNBC	5	2011	8,259	1,624	9,883	17,426	5,128	22,554
TNBC	5	2012	8,465	0	8,465	14,621	0	14,621
TNBC	5	2013	43,873	24,617	68,490	63,622	57,700	121,323
TNBC	5	2014	136,070	1,162	137,232	191,827	2,886	194,713
TNBC	5	2017	167,061	2,467	169,528	201,826	3,262	205,088

*SG= subgroup ,Y=Year,YoD= Year of Diagnosis

© 2022 Jaber Chehayeb R et al. *JAMA Network Open.*

eTable 14. Overall Survival Statistics for the 4 Subgroups and Overall Sample
LCL= Lower confidence limit, UCL= upper confidence limit.

Subgroup	n	events	median	0.95LCL	0.95UCL	Number deleted due to missingness
**HR+HER2-**	9,283	5,700	2.69	2.60	2.78	888
**HR+HER2+**	2,518	1,467	3.13	2.97	3.29	267
**HR-HER2+**	524	433	1.74	1.54	1.95	278
**TNBC**	1,264	1,126	0.877	0.819	0.945	193

© 2022 Jaber Chehayeb R et al. *JAMA Network Open.*

eFigure. Overall Survival Analysis Using Kaplan-Meier Curves by Subgroup

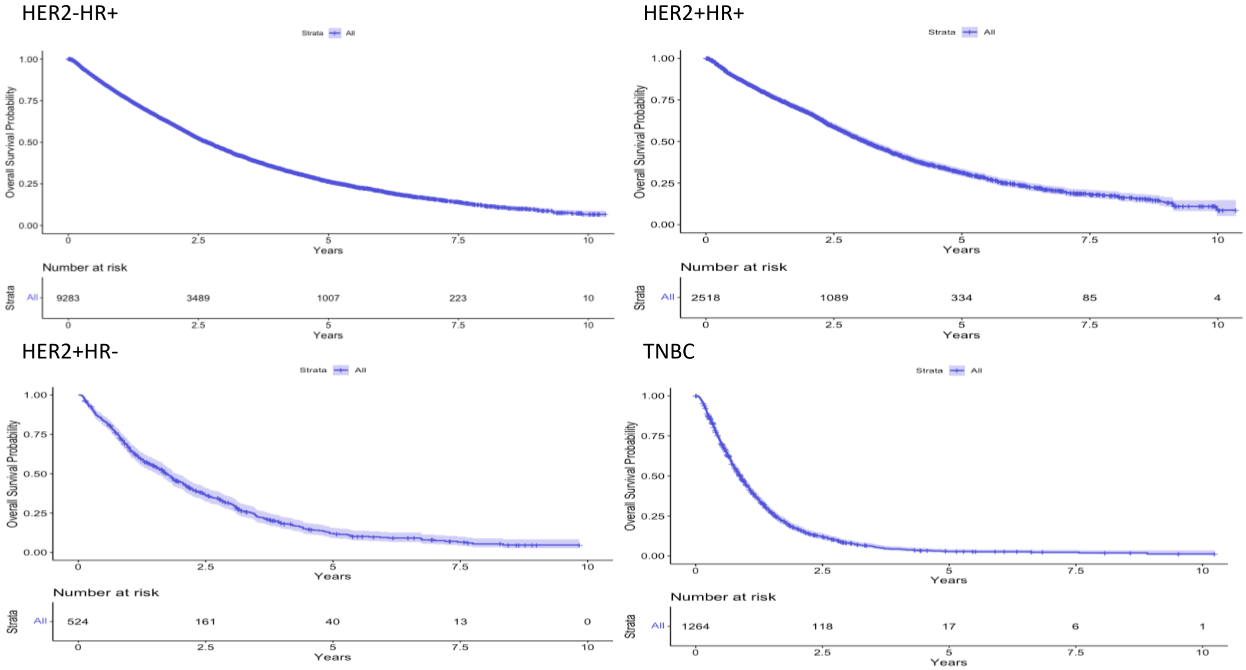

© 2022 Jaber Chehayeb R et al. *JAMA Network Open.*

eReferences

1. Taskindoust M, Thomas SM, Sammons SL, et al. Survival Outcomes Among Patients with Metastatic Breast Cancer: Review of 47,000 Patients. *Ann Surg Oncol.* 2021;28(12):7441-7449.
2. Gobbini E, Ezzalfani M, Dieras V, et al. Time trends of overall survival among metastatic breast cancer patients in the real-life ESME cohort. *Eur J Cancer.* 2018;96:17-24.
3. Zhang L, Li Z, Zhang J, Wu Y, Zhu Y, Tong Z. De novo metastatic breast cancer: Subgroup analysis of molecular subtypes and prognosis. *Oncol Lett.* 2020;19(4):2884-2894.
4. Bartmann C, Wischnewsky M, Stuber T, et al. Pattern of metastatic spread and subcategories of breast cancer. *Arch Gynecol Obstet.* 2017;295(1):211-223.
5. Savci-Heijink CD, Halfwerk H, Hooijer GK, Horlings HM, Wesseling J, van de Vijver MJ. Retrospective analysis of metastatic behaviour of breast cancer subtypes. *Breast Cancer Res Treat.* 2015;150(3):547-557.
6. Wong CR, Njei B, Nguyen MH, Nguyen A, Lim JK. Survival after treatment with curative intent for hepatocellular carcinoma among patients with vs without non-alcoholic fatty liver disease. *Aliment Pharmacol Ther.* 2017;46(11-12):1061-1069.
7. Sundquist M, Brudin L, Tejler G. Improved survival in metastatic breast cancer 1985-2016. *Breast.* 2017;31:46-50.

© 2022 Jaber Chehayeb R et al. *JAMA Network Open.*
