# Supplementary material for: Treatment Sequencing Patterns and Associated Direct Medical Costs of Metastatic Breast Cancer Care in the United States, 2011 to 2021
Source: JAMA Netw Open. 2022 Nov 29;5(11):e2244204. doi: 10.1001/jamanetworkopen.2022.44204 (PMC9709649; doi:10.1001/jamanetworkopen.2022.44204)
Supplement: Supplement 2. — Data Sharing Statement [file jamanetwopen-e2244204-s002.pdf]

Data Sharing Statement

Jaber Chehayeb. Treatment Sequencing Patterns and Associated Direct Medical Costs of Metastatic Breast Cancer Care in the United States, 2011 to 2021. *JAMA Netw Open*. Published November 29, 2022. doi:10.1001/jamanetworkopen.2022.44204

Data
Data available: No

Additional Information
Explanation for why data not available: The data that support the findings of this study have been originated by Flatiron Health, Inc. De-identified data used in this analysis may be made available upon request and are subject to a license agreement with Flatiron Health; interested researchers should contact <DataAccess@flatiron.com> to determine licensing terms.
